# Bioelectronic Direct Current Stimulation at the Transition Between Reversible and Irreversible Charge Transfer

**DOI:** 10.1002/advs.202306244

**Published:** 2024-03-09

**Authors:** Lukas Matter, Oliya S. Abdullaeva, Sebastian Shaner, José Leal, Maria Asplund

**Affiliations:** ^1^ Department of Microtechnology and Nanoscience Chalmers University of Technology Gothenburg SE 41296 Sweden; ^2^ Department of Microsystems Engineering University of Freiburg Georges‐Köhler‐Allee 201 79110 Freiburg Germany; ^3^ Brainlinks‐Braintools Center University of Freiburg Georges‐Köhler‐Allee 201 79110 Freiburg Germany; ^4^ Freiburg Institute for Advanced Studies (FRIAS) University of Freiburg Albertstraße 19 79104 Freiburg Germany; ^5^ Division of Nursing and Medical Technology Luleå University of Technology Luleå SE 97187 Sweden

**Keywords:** capacitance estimation, charge transfer mechanisms, direct current electric fields, reactive oxygen species

## Abstract

Many biological processes rely on endogenous electric fields (EFs), including tissue regeneration, cell development, wound healing, and cancer metastasis. Mimicking these biological EFs by applying external direct current stimulation (DCS) is therefore the key to many new therapeutic strategies. During DCS, the charge transfer from electrode to tissue relies on a combination of reversible and irreversible electrochemical processes, which may generate toxic or bio‐altering substances, including metal ions and reactive oxygen species (ROS). Poly(3,4‐ethylenedioxythiophene) (PEDOT) based electrodes are emerging as suitable candidates for DCS to improve biocompatibility compared to metals. This work addresses whether PEDOT electrodes can be tailored to favor reversible biocompatible charge transfer. To this end, different PEDOT formulations and their respective back electrodes are studied using cyclic voltammetry, chronopotentiometry, and direct measurements of H_2_O_2_ and O_2_. This combination of electrochemical methods sheds light on the time dynamics of reversible and irreversible charge transfer and the relationship between capacitance and ROS generation. The results presented here show that although all electrode materials investigated generate ROS, the onset of ROS can be delayed by increasing the electrode's capacitance via PEDOT coating, which has implications for future bioelectronic devices that allow longer reversibly driven pulse durations during DCS.

## Graphical abstract

Direct current electric fields (dcEFs) naturally occur in wound healing or cancer metastasis. The stimulus to generate dcEFs may release toxic stimulation by‐products. The release can be delayed by increasing the capacitance of the stimulation electrode by coating the surface with conducting polymers.

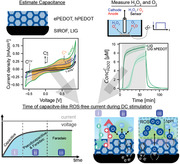

## 1 Introduction

The emergence of electrode materials with enhanced biocompatibility, stability, and performance has contributed to the advancement of bioelectronics.^[^ ^1^ ^]^ In particular, integrating conducting polymers (CPs) as electrode coating materials has ushered in a new generation of organic bioelectronic devices.^[^ ^2^, ^3^, ^4^, ^5^ ^]^ Numerous studies have demonstrated the immense possibilities of using CPs in a wide range of applications, such as biosensors, tissue engineering, drug delivery, and neuromodulation.^[^ ^6^, ^7^, ^8^, ^9^, ^10^ ^]^ Recently, CPs have attracted attention as suitable candidates for organic bioelectronic devices that must deliver direct current (DC).^[^ ^11^, ^12^, ^13^ ^]^ Direct current stimulation (DCS) was found effective in a wide variety of applications, including the activation of neuronal circuits, the regeneration of axons, the control of transgene expression to release hormones, and the promotion of osseointegration in bone anchoring implants.^[^ ^14^, ^15^, ^16^, ^17^ ^]^ However, sustained application of DCS requires specialized electrode materials or technological solutions that mediate between two seemingly contradictory aspects of continuous charge injection namely eluting ions from the electrode but by mechanisms that do not corrode or degrade the electrode or generate toxic concentrations of stimulation by‐products in the tissue.^[^ ^18^, ^19^, ^20^, ^21^, ^22^, ^23^, ^24^ ^]^

Recent research highlights the capability of poly(3,4‐ethylenedioxythiophene) polystyrene sulfonate (PEDOT:PSS) to drive wound healing and electrotaxis in cancer cells, for instance, and underscores the potential of CPs to deliver DCS and modulate biological processes that involve naturally occurring direct current electric fields (dcEFs).^[^ ^25^, ^26^, ^27^, ^28^ ^]^ While these experiments demonstrate the efficiency of DCS within carefully chosen parameters, faradaic charge transfer and its effect on cells and tissue is yet to be taken into account. DCS in itself can influence physiological responses sensitive to reactive oxygen species (ROS), as recently demonstrated in a study on H_2_O_2_‐mediated ion channel activation during DC injection via a PEDOT:PSS/Ti pixel.^[^ ^29^ ^]^ Physiological levels of ROS by‐products have the potential to trigger certain biological signaling pathways without any cytotoxic effects, but could in other applications be a serious concern.^[^ ^29^, ^30^, ^31^, ^32^, ^33^, ^34^ ^]^ The studies listed above showcase that CPs (i.e., PEDOT:PSS) support capacitive as well as faradaic charge injection mechanisms at the electrode‐target (i.e., cell medium, tissue) interface during DCS, and likely can be tailored to support completely biocompatible DCS.^[^ ^13^ ^]^

**Figure 1:**
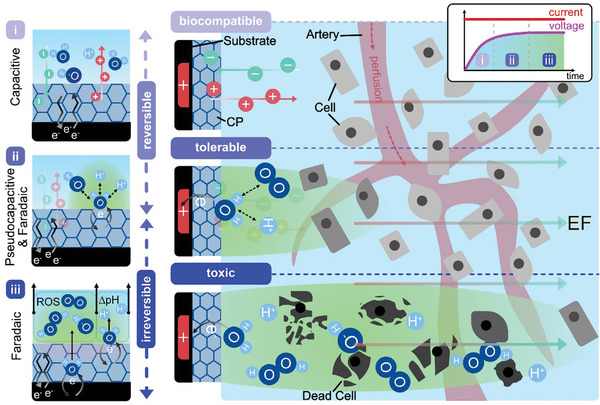
Charge exchange during DCS is based on capacitive (i), pseudocapacitive (ii), and faradaic processes (iii). Faradaic charge injection is reversible, when reaction products are bound (pseudocapacitive) or close the electrode's surface as they can react to their initial form when the current polarity is reversed. Faradaic reactions can lead to irreversible toxic reaction products that diffuse into the target, are volatile, or are involved in secondary reactions. Apart from faradaic reactions directly involving the electrode material, reactions of chemical species present in the biological electrolyte at high concentrations catalyzed by the electrode material are regarded as significant contributors to irreversible charge exchange (see ORR and water electrolysis reactions leading to ROS and a change in pH in Table 1). With time, the main contribution to charge transfer during DCS shifts from reversible to irreversible reactions. The recorded voltage profile (insert, top right) can inform about the nature of the primary charge‐injecting reaction (plateaued voltage indicates faradaic reactions). The generation of toxic and volatile stimulation by‐products is counter‐balanced by internal buffer systems of the body (e.g., enzymatic and chemical buffers) and tissue perfusion.

The charge exchange mechanisms at the electrode‐target interface can be divided into capacitive, pseudocapacitive, and faradaic reactions (as illustrated in **Figure** **1**).^[^ ^35^, ^36^, ^37^ ^]^ Capacitive charge transfer is associated with the redistribution of ions through the formation of an electrical double layer (EDL) and does not involve the transfer of electrons over the electrode‐target interface. Pseudocapacitive charge transfer is driven by faradaic surface reactions primarily enabled by intercalation or adsorption of ions at the surface of the electrode (i.e., porous electrodes, metal oxides).^[^ ^38^, ^39^ ^]^ Faradaic reactions, categorized by the ratio of electron transfer to mass transport rates, can be reversible or irreversible.^[^ ^40^, ^41^ ^]^ Reversible faradaic reactions have a fast electron transfer rate compared to the mass transport rate and generate reaction products that are bound (pseudocapacitive) or close to the electrode's surface. Faradaic reactions with a slow electron transfer rate related to the mass transport rate are irreversible because reaction products diffuse into the target and are unavailable when the pulse polarity is reversed.^[^ ^42^ ^]^ In addition, reactions that lead to products that are involved in secondary reactions (e.g., oxidation of organics) or are volatile (e.g., ROS) belong to the group of irreversible reactions. In general, faradaic reactions inject new charge carriers into the target, while capacitive mechanisms alter the local concentration of ions through redistribution. In DCS, reversible and irreversible processes contribute to charge transfer, although over time, the main charge transfer mechanism shifts from reversible to irreversible (see voltage profile in Figure 1). Besides reactions directly involving the electrode material, reactions with water, hydrogen, and oxygen, such as oxygen reduction reactions (ORR) and water electrolysis, are often regarded as the main contributors to irreversible charge exchange (see reactions in **Table** **1**).^[^ ^35^ ^]^ The electrode material can hereby act as an electrocatalyst accelerating the reaction rate or lowering the required reaction activation energy.^[^ ^43^ ^]^ Changes in the pH and the formation of ROS, hydrogen, and oxygen can be toxic if their generation rate surpasses the regulatory capacity of the target provided by mechanisms such as tissue perfusion or buffer systems (e.g., phosphate buffer in saline solution, enzymatic buffers).^[^ ^32^, ^33^, ^34^, ^35^, ^44^, ^45^ ^]^ For safe electrical stimulation it is therefore critical to understand the time dynamics of irreversible reactions to identify a time window for biologically tolerable DCS (see biocompatible, tolerable, and toxic electrical stimulation in Figure 1). In this paper, the term DCS describes a time‐limited current with pulse durations in the range of minutes to hours.

**Table 1:** ORR and water electrolysis reactions eventually contribute to charge exchange during DCS, because reactants are available in high concentrations in biological electrolytes. In this work, the changes in concentrations of H_2_O_2_ and O_2_ at the electrode's surface are used as markers for the time dynamics of reversible and irreversible electrochemical reactions during DCS.

ORR	O_2_ + 2H^+^ + 2e^−^ → H_2_O_2_	(1)
	O_2_ + 4H^+^ + 4e^−^ **→** 2H_2_O	(2)
	H_2_O_2_ + 2H^+^ + 2e^−^ **→** 2H_2_O	(3)
Water electrolysis	2H_2_O + 2e^−^ **→** H_2_ + 2OH^−^ (reduction)	(4)
	2H_2_O **→** O_2_ + 4H^+^ + 4e^−^ (oxidation)	(5)

Although CPs offer a versatile platform for designing electrode materials, optimizing them for a specific use and tailoring them to match a desired trade‐off between reversible and irreversible charge transfer during DCS remains a challenge and necessitates a detailed knowledge of the charge transfer mechanisms at the electrode‐target interface. To guide the design and selection of an appropriate CP material for a specific application, we require the development of accurate electrochemical tools that identify and quantify the contributions of reversible and irreversible reactions during DCS. This is where our present work comes in, where we characterize CP materials and their respective back electrodes by utilizing a battery of electrochemical measurement techniques such as cyclic voltammetry (CV), chronopotentiometry, and amperometry of stimulation by‐products from irreversible electrochemical reactions during DCS. We focus here on real‐time chemical markers for ORR and water electrolysis. To this end, we utilized commercially available micro O_2_ and H_2_O_2_ sensors, which are faraday‐shielded and incorporate a reference electrode. As H_2_O_2_ and O_2_ play an essential role in different biological processes it is vital to determine if and how much H_2_O_2_ is generated and how H_2_O_2_ generation influences local O_2_ concentration levels. Very high H_2_O_2_ concentrations can cause cell death or interrupt the functionality of cells whereas too low O_2_ concentrations can result in hypoxic conditions.^[^ ^32^, ^33^ ^]^ Measurements of the pH would extend the dataset, but a suitable pH sensor fulfilling the requirements (i.e., faraday‐shielded, smaller tip than tested electrodes) was not available at the time of this work. Nonetheless, we measured the pH with a colorimetric method (polyaniline) in unbuffered saline solution (Figure S1, Supporting Information). We apply these electrochemical tools not only to determine the electrode capacitance, but also to investigate the relationship between electrode capacitance and the time of main charge transfer via reversible mechanisms (i.e., EDL and pseudocapacitance).

In particular, we aim to understand if and how electrode capacitance and material influences the onset of irreversible charge transfer reactions and whether ROS generation can be delayed by increasing the electrode's capacitance via CP coating and prolonging the time of the capacitive‐like current, which would be essential for applications where DC needs to be delivered predominantly by capacitive means and where locally elevated ROS concentration would not be tolerated by the tissue. We focus on CP coating as a method to increase the electrode's capacitance instead of enlarging the electrode's area because the available space is often limited in bioelectronic applications. We use the term capacitive‐like current in this work to refer to the current generated by reversible reactions, which lead to a changing voltage profile over time (comparable to a capacitor's charging/discharging curve). To this end, we benchmark the electrode materials using standard methods in literature to estimate the contribution of reversible and irreversible reactions to the electrode capacitance (i.e., Lindquist, Trasatti, and Dunn method^[^ ^46^, ^47^, ^48^ ^]^), use H_2_O_2_ and O_2_ concentrations as markers for the onset of irreversible electrochemical reactions and assess the correlation between electrode capacitance and the onset of ROS evolution. A recent study triggered more questions about how the formulation of a CP material and the type of back electrode material influence the balance between reversible and irreversible charge transfer.^[^ ^13^ ^]^ Therefore, we compare two CP formulation types, namely electropolymerized PEDOT:PSS (ePEDOT) on sputtered iridium oxide films (SIROF) and hydrogel PEDOT:PSS (hPEDOT) on laser‐induced graphene (LIG) electrodes, as model systems in this work and systematically test how they enhance the capacitive performance of their respective back electrodes and affect ROS generation at the cathode and anode during DCS.

Direct amperometric measurements of H_2_O_2_ and O_2_ concentrations have been previously reported to quantify ROS by‐products during electrical stimulation.^[^ ^29^, ^49^ ^]^ However, to our knowledge, our current study is the first to apply this combination of electrochemical techniques to analyze the time dynamics of reversible and irreversible charge exchange during DCS and determine its influence on H_2_O_2_ and O_2_ generation for different types of CP materials. Ehlich et al.^[^ ^49^ ^]^ investigated ORR during constant voltage and alternating current stimulation (i.e., charge transfer by reversible mechanisms) applied to common electrode materials in neurostimulation. It was found that the onset potential of ORR and the concentration of generated H_2_O_2_ as a by‐product depends on the electrode material. The question of charge exchange dynamics during DCS cannot be answered by simply extrapolating these findings. It requires a specific study with longer pulses to determine how long it takes to reach the onset potential of ORR. In addition, the voltage in our study is measured in a 2‐electrode setup which is the standard way to apply DCS in vivo and in clinical settings.^[^ ^50^, ^51^ ^]^

In summary, the results of this work provide information about the reaction dynamics during DCS at an application level and can, therefore, be directly used by other groups to guide their experiments in the emerging field of dcEFs. First, in Sections 2.1, 2.2, and 2.3, the selection of materials is characterized electrochemically employing electrochemical surface area (ECSA) determination and the Lindquist, Trasatti, and Dunn method to derive the electrodes’ capacitances. This is followed in Section 2.4 by a report of the measured H_2_O_2_ and O_2_ concentrations as markers for the onset of irreversible reactions. In Section 2.5, the time for the capacitive current during DCS is estimated and compared with the time dynamics of the measured chemical concentrations.

## 2 Results

### 2.1 Electrochemical Surface Area

**Figure 2:**
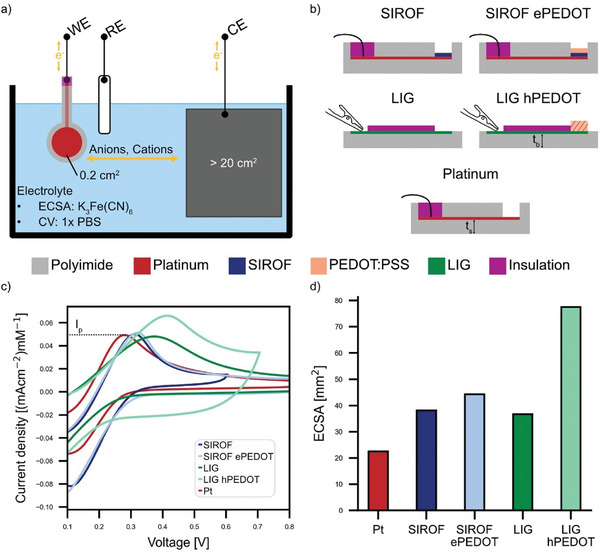
a) Sketch of the experimental setup for electrochemical measurements (CE: Counter electrode, RE: Reference electrode, WE: Working electrode). b) Side view of the investigated electrodes (*t_s_* ≈5 µm, *t_b_* ≈125 µm). c) Anodic peak current (*I_p_*) in K_3_Fe(CN)_6_ solution at 10 mV s^−1^ is increased after coating SIROF and LIG with ePEDOT and hPEDOT, respectively. An increase in peak current implies an enhancement of electron transfer capacity. d) Electrochemical surface area (ECSA) derived from the Randles–Sevcik equation.^[^ ^42^ ^]^

The ECSA is experimentally determined to inform about the density of surface‐active centers at which electrochemical reactions occur. We apply PEDOT coating to enhance the ECSA of the respective base electrode and thus the EDL capacitance. ECSA is further considered as a normalization factor of the applied current density. CVs at different scan rates (*v*) in potassium ferricyanide (K_3_Fe(CN)_6_) are performed to evaluate the ECSA and the electron transfer capacity of the electrodes (**Figure** **2**a). Figure 2b shows the cross‐sectional outline of the investigated electrodes (see Figure S2 for a picture of the electrodes, Supporting Information). All electrodes were designed to have an identical geometrical surface area (*GSA* = 20 mm^2^). The coating of LIG with hPEDOT enhances the electron transfer (one‐electron transfer of ferri/ferrocyanide couple) as shown by the higher anodic peak current (*I_p_*) for LIG hPEDOT compared to bare LIG (Figure 2c). *I_p_* for Pt and SIROF are of comparable magnitude at the presented scan rate (*v* = 10 mVs^−1^), whereas the peak of SIROF ePEDOT is slightly higher, which becomes more pronounced at faster *v* (see full range CVs in Figures S3,S4, Supporting Information). This suggests that both types of PEDOT coatings improve the electron transfer capability of the base electrode. The ECSA of Pt, SIROF, SIROF ePEDOT, LIG, and LIG hPEDOT electrodes is 22.4, 38.1, 44.3, 36.7, and 77.5 mm^2^, respectively (Figure 2d). As expected, the PEDOT coating enhances the ECSA, and hPEDOT coating more than doubles the ECSA of LIG.^[^ ^13^, ^52^ ^]^ In comparison, the increase in ECSA by coating SIROF with ePEDOT is modest at ≈15%. ePEDOT coating is a common method for microelectrodes while hPEDOT is usually applied at macroelectrodes. In this work, we compare both PEDOT coating methods with typical base electrodes to lay the foundation for a wide variety of applications (e.g., in cell culture, neurostimulation and electrode–skin interfaces).

### 2.2 Analysis of Cyclic Voltammograms

CVs are analyzed to classify the materials as capacitive, pseudocapacitive, or battery‐like. In response to CV, capacitive materials show a potential‐independent current, meaning that the current plateaus during the voltage scan in each direction. In contrast, battery‐like materials have distinct redox current peaks. Pseudocapacitive materials show features of both categories, meaning a potential‐independent current superimposed with peaks.^[^ ^40^, ^41^ ^]^ The report of the electrode capacitance is only suggested for capacitive and pseudocapacitive materials because capacitance ([Farad]) indicates a linear dependence of the stored energy and the range of the voltage window. Battery‐like materials’ capacity ([Ah]) should be indicated instead because the charge is mainly stored in a narrow voltage window.^[^ ^36^, ^39^ ^]^ This work investigates the relationship between electrode capacitance and ROS‐free capacitive‐like current delivery time. Therefore, this section lays the foundation for further estimating the electrode capacitances.

**Figure 3:**
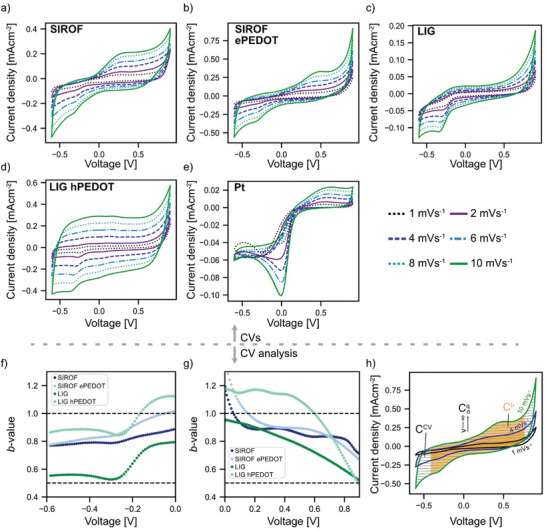
a) CVs of SIROF. b) SIROF ePEDOT. c) LIG. d) LIG hPEDOT. e) Pt. All in 1× PBS. f) *b*‐values according to the Lindquist method^[^ ^46^ ^]^ for the cathodic voltage window show a drop at −0.3 V, which describes the peak in the CVs. *b*‐values decrease toward lower voltages. g) The *b*‐values for the anodic CV voltage window decrease toward higher voltages indicating a transition from surface‐controlled to diffusion‐controlled reactions. h) Sketch of the methods applied to estimate capacitance. $C^{CV}$ and $C^{b}$ are calculated from the charge during a CV cycle, whereas the voltage limits for $C^{b}$ are adjusted to include only capacitive‐like currents (*b*‐value ≥ 0.8). $C_{o}^{q}$ (Trasatti method) is the fitted *y*‐intercept (*v* → ∞) of the linear regression of the *C* versus *v* curve (*v* = 1–10 mV s^−1^).

**Figure** **3**a–e shows *v*‐dependent CVs of the investigated materials in 1× phosphate‐buffered saline (PBS). At the anodic limit of the voltage window, SIROF‐ and LIG‐based materials show an increasing current density. Except for LIG hPEDOT and Pt, the current density also rises toward the cathodic voltage limit. The voltage window is chosen within the so‐called “water window” (−0.6 to 0.9 V vs Ag/AgCl) with the anodic and cathodic limits in the range of the onset of water electrolysis, although we are aware that the exact boundaries are electrode material specific.^[^ ^53^ ^]^ A clear reduction peak at 0 V is observed for Pt, with a less pronounced oxidation peak at 0.6 V. The other materials show a reduction peak at −0.3 V, but only SIROF and SIROF ePEDOT show oxidation peaks at 0.25 and 0.4 V, respectively. The CV profiles of LIG, SIROF, SIROF ePEDOT, and LIG hPEDOT show a clear capacitive envelope with potential‐independent currents at faster *v* (*v* > 10 mV s^−1^, Figure S6, Supporting Information). For slower *v* (*v* ≤ 10 mV s^−1^), potential‐independent currents are only apparent for LIG hPEDOT, whereas the other materials show a potential‐independent current only in a subset of the investigated voltage range (e.g., ≈ −0.25 to 0.5 V for LIG).

Reaction kinetics were further investigated by fitting the recorded currents *I* at different *v* to calculate *b*‐values according to the Lindquist method^[^ ^46^ ^]^

(1) $$I=a\cdot v^{b}$$

where *a* and *b* are adjustable parameters, which are determined from the log(*I*) versus log(*v*) plot. According to theory, the current of electrochemical reactions scales with the square root of *v* for diffusion‐controlled reactions (*b* = 0.5) (e.g., metal redox, irreversible reactions), while it scales linearly for surface‐controlled mechanisms (*b* = 1) (e.g., EDL, surface redox, reversible processes). We mainly focus on peaks in CVs to reveal the underlying electrochemical processes. In the cathodic voltage window, *b*‐values of SIROF, SIROF ePEDOT, and LIG hPEDOT lay between 1.1 and 0.8 (Figure 3f). Thus, the *b*‐values of the underlying electrochemical reactions point toward reversible mechanisms. The *b*‐values of LIG are in the range of 0.7 to 0.5, speaking for a mix of reversible and irreversible processes. All materials have a dip in their *b*‐value curve at −0.3 V because of the peak in the CVs. The dip is more pronounced for LIG and LIG hPEDOT than for the other materials tested. However, only LIG *b*‐values are close to 0.5 through the dip. At the lower limit of the voltage window, *b*‐values for LIG and LIG hPEDOT plateau at 0.55 and 0.85, respectively, while they decrease for SIROF and SIROF ePEDOT. The decrease in *b*‐values toward the limits of the water window is also shown in the *b*‐values of the anodic voltage window (Figure 3g). Here, *b*‐values for LIG and LIG hPEDOT at 0.9 V are 0.5, indicating the onset of irreversible electrochemical reactions. For LIG, *b*‐values decrease nearly linearly from 1 to 0.5. The *b*‐values for SIROF‐based electrodes and LIG hPEDOT plateau at 0.9 and 1.2 between 0.2 and 0.6 V and 0 and 0.5 V, respectively. The peaks in the CVs of SIROF and SIROF ePEDOT at 0.25 and 0.4 V, respectively, do not lead to a decrease in *b*‐values, speaking for mainly reversible processes in that voltage range.

According to the analysis of CVs and *b*‐values, SIROF, SIROF ePEDOT, LIG, and LIG hPEDOT belong to the group of pseudocapacitive materials because of a potential‐independent current during voltage sweeps and *b*‐values ≈1. The latter statement is only valid in a material specific subset of the investigated voltage range (i.e., reversible *b*‐value plateaus), which is considered in Section 2.3 when estimating the electrode capacitances. The calculation of *b*‐values for Pt resulted in linear fits with R‐values below the acceptance limit of 0.95 (see R‐values in supplementary). Therefore, we leave the analysis of *b*‐values for the whole voltage range out for Pt. At the prominent oxide reduction peak at 0 V in the CVs of Pt the *b*‐value is below 0.5. In addition, Pt's CVs do not show a noteworthy voltage range with potential‐independent currents. Thus, Faradaic reactions have a high contribution to the charge storage of Pt leading to its classification as battery‐like material.

### 2.3 Estimation of Electrode Capacitance

The recorded current during cyclic voltammetry can be divided into a surface‐controlled and diffusion‐controlled portion. Surface‐controlled mechanisms lead to capacitive‐like currents and are highly reversible because reaction products are bound to the surface and, thus, are available for the counter reaction when the current polarity is changed and are less likely to participate in secondary reactions.^[^ ^42^ ^]^ A larger capacitance based on reversible mechanisms should translate to a longer time of capacitive‐like ROS‐free DCS. This section estimates the capacitance according to methods commonly used in the literature to assess the contribution of reversible and irreversible reactions to the electrode's capacitance.^[^ ^46^, ^47^, ^48^, ^54^, ^55^, ^56^, ^57^ ^]^ The capacitance estimation methods are based on CVs at different *v* in 1× PBS, as the current responses to slow and fast voltage scans give insights into the reversibility of an electrochemical reaction (e.g., in the context of DCS diffused reaction products are not available for the counter reaction).

The CV method uses the whole voltage range to estimate the capacitance based on the delivered charge during CV ($C^{CV}$) (see Figure 3h for their definition) which is also known as the charge storage capacity in bioelectronics.^[^ ^53^ ^]^ CV itself does not exclude irreversible reactions. Thus, following the idea of Lindquist to distinguish the nature of the electrochemical process by their *b*‐values, we estimate the capacitance $C^{b}$ by only considering the voltage window of the recorded CVs with *b*‐values ≥ 0.8 (i.e., mainly reversible mechanisms). We have chosen the threshold according to the theory that a spherical diffusion process yields a *b*‐value of 0.75; thus, the threshold to exclude irreversible reactions should be higher.^[^ ^42^ ^]^ The Trasatti method^[^ ^48^ ^]^ can be used to calculate a bulk (i.e., irreversible reactions) and an outer surface capacitance (i.e., reversible mechanisms). We report the outer surface capacitance because it excludes irreversible reactions. The outer surface capacitance, according to Trasatti $\left(C_{o}^{q}\right)$ is the capacitance calculated from the charge enclosed by the CV when *v* goes to infinite (see Figure 3h). Dunn argued that the current at each potential in a CV consists of a diffusion‐controlled and a capacitive‐like current. Therefore, the fraction of the current during CV that is capacitive‐like can be estimated, resulting in a capacitance value. However, applying the Dunn method,^[^ ^47^ ^]^ we observed capacitive‐like currents of higher magnitude than the recorded currents (Figures S7,S8, Supporting Information). In the Dunn method the recorded current is described by

(2) $$I=k_{1}\;v+k_{2}v^{1/2}$$

where *k*_1_ and *k*_2_ are fitting parameters. We calculated *b*‐values (Figure 3f,g) above 1 that cannot be accurately fitted by the assumptions of the Dunn method.^[^ ^55^ ^]^ Thus, we conclude that the Dunn method is unsuitable for analyzing the recorded CVs, which led us to exclude the capacitances calculated with this method from further analysis in this work. CVs of Pt are not considered because of the distinct redox peaks in the CV, which means that charge storage is mainly available in the narrow window of the peaks with negligible capacitive envelope.

**Table** **2** shows the results for $C^{CV}$, $C^{b}$ and $C_{o}^{q}$. $C^{CV}$ and $C^{b}$ are not notably different. Since this work focuses on DCS, we emphasize the results for slow *v* here, while estimated capacitances for a wider scan rate window are shown in Figure S9 (Supporting Information). For slow scan rates (*v* ≤ 10 mV s^−1^), $C^{CV}$ and $C^{b}$ decrease linearly with *v*, followed by a nonlinear range (*v* > 10 mV s^−1^) (Figure S9, Supporting Information). The former is due to less time for ions to diffuse with increasing *v*, while the latter is likely due to the uncompensated ohmic drop and irreversibility of redox reactions.^[^ ^54^, ^56^, ^57^ ^]^ In contrast, $C^{CV}$ and $C^{b}$ of LIG hPEDOT increase as *v* is reduced which is not expected. Scan rates from 1 to 10 mV s^−1^ were considered for calculating $C_{o}^{q}$ as the curves obtained were only linear in this reduced scan rate window (Figure S10, Supporting Information). $C_{o}^{q}$ is different from $C^{b}$(1 mV s^−1^). For SIROF, SIROF ePEDOT, and LIG, $C^{b}$ (1 mV s^−1^) is smaller than $C_{o}^{q}$, whereas for LIG hPEDOT it is larger. In general, and as is also expected, the PEDOT coating enhances the estimated capacitance for both SIROF and LIG as the underlying electrode material, and this is regardless of the method used to estimate the capacitance.

**Table 2:** Capacitance according to CV ($C^{CV}$), Lindquist ($C^{b}$), and Trasatti ($C_{o}^{q}$) method for LIG, LIG hPEDOT, SIROF, and SIROF ePEDOT.

	$C^{CV}$(1 mV s^−1^)	$C^{CV}$(10 mV s^−1^)	$C^{b}$(1 mV s^−1^)	$C^{b}$(10 mV s^−1^)	$C_{o}^{q}$
	[mFcm^−2^]				
LIG	6.12	3.03	5.24	2.50	1.63
LIG hPEDOT	14.68	24.42	13.62	26.14	30.53
SIROF	20.34	11.62	21.86	11.97	8.30
SIROF ePEDOT	22.76	14.81	22.53	15.19	10.63

### 2.4 Measurement of H_2_O_2_ and O_2_ During Direct Current Stimulation

**Figure 4:**
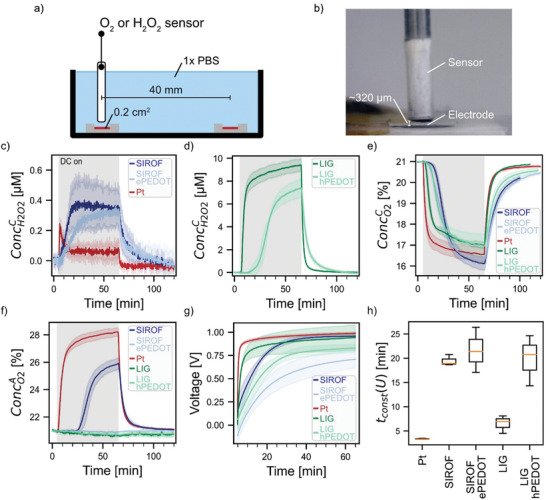
a) Sketch of the measurement setup. Two 20 mm^2^ electrodes acting as cathode and anode are separated by 40 mm in 1× PBS. b) The O_2_ or H_2_O_2_ sensor was positioned ≈320 µm above the center of the electrode surface. H_2_O_2_ generation and O_2_ generation and consumption were measured in three independent experiments (three samples per experiment): H_2_O_2_ sensor at the cathode, O_2_ sensor at the anode, and O_2_ sensor at the cathode. c–f) Measured H_2_O_2_ concentration at the cathode ($Conc_{H2O2}^{C}$) and O_2_ concentration at the anode ($Conc_{O2}^{A}$) and the cathode ($Conc_{O2}^{C}$) during DCS (1 h, 10 µA cm^−2^). No stimulation was applied for the first 5 min. g) Recorded voltage during 1 h DCS. h) Time until the recorded voltage is quasi‐constant (*t_const_* (*U*)). Solid lines shown in (c–g) represent H_2_O_2_ and O_2_ concentration traces. Shaded areas represent standard deviation (SD).

The calculated reversible electrode capacitance, which is based on surface‐controlled mechanisms should theoretically correlate with the time of capacitive‐like ROS‐free current possible during DCS. In other words, the time over which DC is mainly provided by reversible mechanisms. Apart from reactions directly involving material from the electrode, reactions with water, hydrogen, and oxygen are typically considered the major contributors to irreversible charge transfer in bioelectronic stimulation.^[^ ^35^ ^]^ Therefore, we used products generated during ORR and water electrolysis (see reactions in Table 1) as markers for the onset of the main charge transfer by irreversible reactions to evaluate the time of capacitive‐like ROS‐free current. We experimentally measured the H_2_O_2_ concentration at the cathode ($Conc_{H2O2}^{C}$) and the O_2_ concentration at the cathode ($Conc_{O2}^{C}$) and the anode ($Conc_{O2}^{A}$) (the setup is shown in **Figure** **4**a,b) during DCS (1 h, 10 µA cm^−2^). O_2_ at the cathode can be reduced in a two‐ or four‐electron ORR to either H_2_O_2_ (Reaction (1) in Table 1) or H_2_O (Reaction (2)), respectively. Generated H_2_O_2_ can further react to H_2_O (Reaction (3)).^[^ ^58^ ^]^ It has to be noted that H_2_O_2_ and O_2_ have long lifetime and are cell membrane‐diffusible end products of electrochemical reactions, which consequently have the capacity to spread in the target.^[^ ^59^ ^]^ In intermediate steps other ROS than H_2_O_2_ and O_2_ may be generated (e.g., peroxide and superoxide radicals).^[^ ^58^ ^]^ With the sensors used in this work, it is not possible to measure the concentration of these intermediate reaction products.

SIROF, SIROF ePEDOT, and Pt generate a small amount of H_2_O_2_ compared to LIG and LIG hPEDOT (Figure 4c,d). ePEDOT coating delays the rise of $Conc_{H2O2}^{C}$ which eventually reaches the same value measured at uncoated SIROF electrodes after 1 h DCS. hPEDOT coating delays the onset of H_2_O_2_ generation at the cathode compared to pristine LIG. All investigated materials consume O_2_ at the cathode (Figure 4e). LIG consumes O_2_ and releases H_2_O_2_ earlier than LIG hPEDOT. SIROF ePEDOT consumes O_2_ earlier than pristine SIROF. Further discussion on the time dynamics of the measured concentration changes and their correlation to the electrode capacitances is included in Section 2.5. SIROF‐ and Pt‐based electrodes consume the most and second most amount of O_2_ but only generate comparably small amounts of H_2_O_2_ indicating that O_2_ is utilized in other electrochemical reactions than the two‐electron ORR such as the four‐electron ORR. It is likely that LIG electrocatalyses the two‐electron ORR, as shown by the high amount of generated H_2_O_2_. The electrocatalytic activity of LIG toward the two‐electron ORR is decreased by coating LIG with hPEDOT, evidenced by a comparable change in $Conc_{O2}^{C}$ but a smaller change in $Conc_{H2O2}^{C}$ after 35 min of DCS. Out of all materials tested, only SIROF and Pt release O_2_ at the anode (Figure 4f), which interestingly is prevented after coating SIROF with ePEDOT. We were further interested in the time of capacitive‐like current when multiple DC pulses are applied instead of 1 h DCS. We thus divided the 1 h DCS into 4 monophasic consecutive 15 min pulses, with a 15 min inter‐pulse period, and measured $Conc_{H2O2}^{C}$. As shown in Figure S11 (Supporting Information), the repeated pulsing polarizes the electrodes, which decreases the time of capacitive‐like current in consecutive pulses.

### 2.5 Capacitive Current Time

The recorded voltage during DCS increases because the charge transfer rate from the simultaneously happening electrochemical reactions needs to be in balance with the electron transfer rate (*i.e*., the applied current). First in time, the charging of the EDL leads to an exponential rise in the recorded voltage, because the load of this capacitance will increase when it is charged. Pseudocapacitive mechanisms such as the expulsion/incorporation of ions in the CP add a second opportunity for charge transfer maintaining a lower voltage for longer. The recorded voltage plateaus when irreversible faradaic reactions (i.e., water splitting) dominate the charge transfer.^[^ ^13^ ^]^ The recorded voltage from the DCS was analyzed during the measurement of $Conc_{H2O2}^{C}$ (Figure 4g) to estimate the time window in which the capacitive‐like current dominates. *t_const_* (*U*) is the time after which the recorded voltage is quasi‐constant (c.f. Methods). The results show that *t_const_* (*U*) is less than 5 min for Pt, ≈6 min for LIG, and more than 15 min for LIG hPEDOT, SIROF, and SIROF ePEDOT (Figure 4h). The most striking result is that the PEDOT coating substantially increased *t_const_* (*U*) for LIG.

In order to correlate the measured concentrations to *t_const_* (*U*) and the estimated capacitance, we determined the time points after which the measured concentrations reached 1% and 50% of their respective maximum/minimum (*t*_1_(*Conc*) and *t*_50_(*Conc*)). The former is assigned to describe the onset of the measured irreversible reactions and the latter the time after which the underlying electrochemical reactions have reached considerable rates. For comparison, the various metrics are shown in **Table** **3**. $C^{b}$(1 mV s^−1^) is listed instead of $C^{CV}$ and $C_{o}^{q}$ because it is calculated using similar methods as the most common electrode capacitance metric in bioelectronic research ($C^{CV}$) but adjusted to exclude irreversible reactions. For Pt and LIG, H_2_O_2_ generation and O_2_ consumption follow similar time dynamics (i.e., $t_{1}\left(Conc_{H2O2}^{C}\right)$≈$t_{1}\left(Conc_{O2}^{C}\right)$) (Figure 4c,e). Coating LIG with hPEDOT substantially increases $C^{b}$ (1 mV s^−1^) and $t_{1}\left(Conc_{H2O2}^{C}\right)$, which is ≈3× larger than $t_{1}\left(Conc_{O2}^{C}\right)$ (Figure 4d,e). Thus, for LIG hPEDOT, O_2_ is consumed earlier than H_2_O_2_ is generated indicating that other O_2_‐consuming electrochemical reactions besides the two‐electron ORR contribute to charge transfer (e.g., four‐electron ORR). In this intermediate period of O_2_ consumption without H_2_O_2_ generation, there is an increase in the recorded voltage, leading us to expect that reversible mechanisms are contributing to charge transfer. The reversible mechanisms are exhausted later for LIG hPEDOT and like pristine LIG, a constant voltage is reached after $t_{1}\left(Conc_{H2O2}^{C}\right)$ (Figure 4g). For SIROF and SIROF ePEDOT, the two‐electron ORR does not substantially contribute to charge transfer because H_2_O_2_ is generated in small concentrations. Moreover, $C^{b}$ (1 mV s^−1^) and *t_const_* (*U*) are slightly increased for SIROF ePEDOT compared to SIROF. The recorded voltage for SIROF turns constant shortly after $t_{1}\left(Conc_{O2}^{A}\right)$ (0.8 min), marking the transition from capacitive‐like current, to current delivery relying on irreversible reactions (i.e., water oxidation). In this instance, the slope of the recorded voltage is a valuable indicator for the shift of charge transfer from mainly reversible to irreversible reactions. For all materials, the O_2_ consumption increases notably during the quasi‐constant recorded voltage. For Pt, LIG, and SIROF $t_{50}\left(Conc_{O2}^{C}\right)$ is within the same range as *t_const_* (*U*). The magnitude of the recorded voltage does not indicate the rate of the underlying electrochemical reaction as evidenced by an increase in O_2_ consumption after *t_const_* (*U*) is reached. By consuming more O_2_, the underlying electrochemical reaction contributes more to the total transferred charge at that time. Therefore, other electrochemical processes need to be exhausted or become energetically less favorable over time which is compensated by an increased rate of the O_2_‐consuming electrochemical reactions. As depicted in Figure 1, DCS should only be implemented in a range where electrochemical by‐products are either not generated or are buffered through homeostatic processes or tissue perfusion. Our results show that charge transfer of the tested electrodes increasingly relies on irreversible reactions, generating ROS and consuming O_2_, when a plateau is reached in the stimulation voltage. However, as irreversible reactions may to that time point not have reached a rate where toxic concentrations of stimulation by‐products are generated, stimulation longer than *t_const_* (*U*) may be within the tolerable range if tissue buffering is taken into account.

**Table 3:** Time to reach 1% and 50% (*t*_1_ and *t*_50_) of the maximum/minimum measured H_2_O_2_ and O_2_ (at anode A and cathode C) concentration for each material. $C^{b}$ (1 mV s^−1^) and the estimated time of capacitive‐like current from voltage excursions during DCS (*t_const_* (*U*)).

	${Conc}_{H2O2}^{C}$		${Conc}_{O2}^{C}$		${Conc}_{O2}^{A}$		$C^{b}$ (1 mV s^−1^) [mF cm^−2^]	*t_const_* (*U*) [min]
	*t*_1_ [min]	*t*_50_ [min]	*t*_1_ [min]	*t*_50_ [min]	*t*_1_ [min]	*t*_50_ [min]		
Pt	0.4	0.6	0.3	2.3	0.8	4.2	/	3.4
LIG	1.6	4.0	2.0	6.7	/	/	5.2	6.5
LIG hPEDOT	10.3	26.9	4.1	14.5	/	/	13.6	19.9
SIROF	0.2	7.9	6.4	19.9	20.2	30.4	21.9	19.4
SIROF ePEDOT	0.3	15.2	3.6	17.8	/	/	22.5	21.6

## 3 Discussion

In this work, we wanted to verify whether increasing the electrode capacitance by coating with a CP (PEDOT) leads to a longer time of capacitive‐like ROS‐free current delivery. For that purpose, we first experimentally determined the ECSA of the electrodes to normalize the applied DC. Second, we applied four methods to analyze CVs to categorize the materials and to estimate the electrodes’ capacitance. We then considered the chemical concentrations of H_2_O_2_ and O_2_ as markers for the onset of irreversible reactions and finally compared the obtained values to draw conclusions.

In all experiments a current density of 10 µA cm^−2^ was applied for 1 h, with the intention of normalizing the current to the geometrical electrode area. The electrodes were stable during the experiments. The excellent stability of the investigated electrodes has previously been shown in repeated CV and pulsing experiments.^[^ ^12^, ^13^, ^52^, ^60^ ^]^ As the ECSA may differ from the geometrical area, we decided also to measure the ECSA to determine the effective current density. We have shown that the ePEDOT and hPEDOT coatings enhance the electron transfer, which is more pronounced for the underlying LIG than for SIROF for all the base electrode and CP combinations investigated in this work (Figure 2c). The increase in ECSA after PEDOT coating is in line with expectations and literature.^[^ ^61^ ^]^ It should be noted that this work compares two different PEDOT:PSS formulations. LIG is coated with hPEDOT whereas SIROF is coated with ePEDOT, creating material combinations recently shown to deliver DCS effectively.^[^ ^12^, ^13^, ^25^, ^26^ ^]^ Although the two PEDOT coatings are closely related, they may differ in thickness (thickness hPEDOT ≈3× ePEDOT),^[^ ^12^, ^52^ ^]^ mechanical properties, electronic conductivity, and porosity, which is essential to keep in mind for the remainder of this discussion. Nevertheless, the fundamental charge transfer mechanisms during DCS are assumed to be comparable for the two material combinations. The ratio of PEDOT to PSS is higher in ePEDOT than in hPEDOT (1.9 vs 0.7), but both formulations consist of the same chemistry and, therefore, offer the same species to be involved in electrochemical reactions.^[^ ^52^ ^]^ A larger ECSA translates to more reaction centers around which the EDL builds up, and electrochemical reactions occur. The effective current densities applied in this study during experimentation are 8.9, 5.2, 4.5, 5.5, and 2.6 µA cm^−2^ for Pt, SIROF, SIROF ePEDOT, LIG, and LIG hPEDOT, respectively (see ECSA in Figure 2d). By coating LIG with hPEDOT, the effective current density is more than halved, which is expected to prolong the time of capacitive‐like current delivery with respect to the geometrical area. By introducing a second material through coating with ePEDOT and hPEDOT, the relationship between ECSA and capacitive‐like current time is not straightforward as other reaction pathways within PEDOT:PSS may also play a role in current delivery. The underlying electrode materials and PEDOT:PSS are porous.^[^ ^13^, ^52^ ^]^ We propose that the electrolyte can easily diffuse through these pores, react differently at the surface or bulk of PEDOT:PSS, and reach the underlying electrode where other electrochemical reactions can occur in parallel.

CVs over a range of *v* (1–300 mV s^−1^) were recorded in 1× PBS to provide information on the electrochemical reaction kinetics. PBS is widely accepted as a medium that mimics the pH, osmolarity and ion concentration of the human body.^[^ ^62^ ^]^ We found that a potential‐independent current is observed in the CVs of SIROF‐ and LIG‐based electrodes which is typical for pseudocapacitive materials (Figure 3a–e and Figure S6, Supporting Information).^[^ ^36^, ^39^ ^]^ In addition to the shape of the CV, we investigated the *b*‐values according to the Lindquist method to categorize the tested materials as capacitive, pseudocapacitive, or battery‐like (Figure 3f,g). The reduction process at −0.3 V can be seen in LIG‐ and SIROF‐based electrodes, indicating a reaction general for all tested electrodes. We hypothesize that the peak is due to hydrogen adsorption,^[^ ^53^, ^63^ ^]^ which has been reported as a pseudocapacitive process for activated carbon.^[^ ^64^ ^]^ The peak at −0.3 V also translates into a drop in *b*‐values for the SIROF‐ and LIG‐based materials in this work, which is not expected for a purely reversible mechanism. In the case of SIROF, SIROF ePEDOT, and LIG hPEDOT, the *b*‐value at −0.3 V is ≈0.8, indicating a mix of reversible and irreversible processes. In the CV of SIROF for *v* > 10 mV s^−1^, the typical peaks of Ir(III)/Ir(IV) transitions are present at 0.25 V (anodic) and 0.2 V (cathodic) (Figure S6c, Supporting Information).^[^ ^65^, ^66^ ^]^ The *b*‐values at these voltages are ≈1, indicating a reversible reaction as previously reported.^[^ ^66^, ^67^, ^68^ ^]^ Coating SIROF with ePEDOT shifts the Ir(III)/Ir(IV) transition potentials to higher voltages. Ir(III)/Ir(IV) transition peaks are less pronounced at faster *v* in SIROF ePEDOT compared to pristine SIROF, showing that capacitive processes of ePEDOT are more dominant for fast charge exchange. To summarize, the analysis of the CVs at different *v* reveals that LIG, SIROF, SIROF ePEDOT, and LIG hPEDOT are pseudocapacitive materials in a limited voltage window because 1) they show a potential‐independent current envelope and 2) a voltage range with *b*‐values ≈1 can be identified.

We considered the measured changes in concentrations of H_2_O_2_ and O_2_ at the electrode's surface during stimulation, as markers for the onset of irreversible reactions. The generation of H_2_O_2_ is due to oxygen reduction, while the release of O_2_ is caused during water electrolysis (see reactions in Table 1).^[^ ^53^, ^58^ ^]^ We have also investigated the change in pH by a colorimetric method, which is less precise (Figure S1, Supporting Information). We have shown that LIG generates a noteworthy amount of H_2_O_2_, while SIROF‐based electrodes and Pt produce only low concentrations of H_2_O_2_ (Figure 4c,d) (ratio maximum $Conc_{H2O2}^{C}$ Pt versus LIG is 0.01). These results agree with the literature, as LIG has recently been reported to be an effective catalyst for the electrochemical synthesis of H_2_O_2_, while SIROF and Pt have been shown to produce a insignificantly small amount of H_2_O_2_ during current delivery.^[^ ^49^, ^69^ ^]^ In fact Pt is known as a metal catalyst for ORR, however other ORR pathways exist which do not lead to H_2_O_2_, e.g. four‐electron reaction pathway resulting in the reduction of oxygen to water.^[^ ^58^ ^]^ Coating SIROF with ePEDOT resulted in a slower increase in $Conc_{H2O2}^{C}$ and the complete inhibition of O_2_ generation during stimulation, while coating LIG with hPEDOT mainly delayed the onset of H_2_O_2_ production (Figure 4d). Previous reports on the electrocatalytic activity of PEDOT concerning H_2_O_2_ generation have been contradictory. PEDOT has been reported as a suitable material to support the electrochemical synthesis of H_2_O_2_, demonstrating the potential of PEDOT to be utilized in applications that require the on‐demand generation of ROS at physiological concentration levels for the modulation of ROS‐dependent physiological processes.^[^ ^29^, ^70^ ^]^ In contrast, it has been shown that coating metal electrodes (i.e., gold) with PEDOT:PSS reduces ROS generation.^[^ ^71^ ^]^ Our experiments add the perspective of the supporting electrode material. Coating LIG with hPEDOT significantly delays the generation of H_2_O_2_. However, the coating does not prevent the electrocatalytic activity of the underlying LIG from generating H_2_O_2_, as the electrolyte may reach the underlying LIG because of the porosity of hPEDOT. Our results show that the underlying electrode material must be considered when quantifying electrochemical reaction products. In addition, both PEDOT formulations either delayed or inhibited the generation of highly volatile reaction products, which is important information as it leaves more leeway for nontoxic DCS, which does not alter the chemical composition of the stimulation target.

To return to the original question, does enhancing the electrode capacitance by PEDOT coating lead to a longer time of capacitive‐like ROS‐free current. According to theory, the relationship between capacitance and time of capacitive‐like current is linear, assuming a single material electrode. The capacitance of a single material electrode can be increased by enlarging its surface area (i.e., increasing roughness or geometrical area), which results in a longer time for the EDL to charge/discharge.^[^ ^42^ ^]^ However, bioelectronic applications frequently encounter spatial constraints, given that implants must conform to confined spaces within the tissue. We consequently explore PEDOT coating as a method to enhance the electrode capacitance, without increasing the geometric surface area of the coated electrode. Since PEDOT offers new reaction pathways, but in the meantime is porous as well and allows electrolyte to reach the underlying substrate, the correlation between capacitance and time of ROS‐free current is unknown. We have shown that coating LIG with hPEDOT triples the capacitance and increases $t_{1}\left(Conc_{H2O2}^{C}\right)$ by a factor of ten (Table 2). SIROF‐based electrodes generate a low concentration of H_2_O_2_, meaning that the underlying electrochemical reaction contributes only to a small extent to the total charge transfer. Based on the delay of H_2_O_2_ evolution after coating LIG with hPEDOT, a similar result could be expected for SIROF ePEDOT. Coating SIROF with ePEDOT only slightly (3%) increases the estimated capacitance (*C_b_* (1 mV s^−1^)). This gradual increase cannot delay $t_{1}\left(Conc_{H2O2}^{C}\right)$ but it slows the reaction rate as seen by a slower rise of the H_2_O_2_ concentration curve (Figure 4c). For Pt, LIG, SIROF, and SIROF ePEDOT the current immediately after $t_{1}\left(Conc_{H2O2}^{C}\right)$ is still capacitive‐like as can be seen from the fact that the recorded voltage has not yet stabilized. This observation divides the initial research question into two parts. A capacitive‐like current does not exclude that irreversible reactions such as H_2_O_2_ evolution contribute to charge transfer. Reversible and irreversible processes occur in parallel with reversible mechanisms mainly contributing to charge transfer in the early stages of DCS. The reversible mechanisms are exhausted with time, and the charge delivery shifts to irreversible reactions. This is consistent with our previous observations and proposed model for electrochemical reactions during DCS with rough substrates and CP coatings.^[^ ^13^ ^]^ We propose that the constant magnitude of the recorded voltage is related to the largest required voltage of the various electrochemical reactions occurring simultaneously. Our data suggest that water oxidation on SIROF is the electrochemical reaction with the largest required voltage because the recorded voltage plateaus shortly after the onset of O_2_ evolution (Table 3). For Pt, LIG, SIROF, and LIG hPEDOT other oxygen‐consuming reactions influence the magnitude of the recorded voltage as seen by a *t_const_* (*U*) greater than $t_{1}\left(Conc_{H2O2}^{C}\right)$ while $Conc_{02}^{C}$ increases. If we consider *t_const_* (*U*) as a marker for the time of the capacitive‐like current, there seems to be a correlation with the estimated capacitances, although it is not linear and only valid between groups with the same base electrode. Qualitatively, it can be said that an increase in capacitance ($C^{b}$ and $C_{o}^{q}$) leads to a larger *t_const_* (*U*) assuming that electrodes with the same substrate are compared.

Based on the observation that capacitance is related to the time of capacitive‐like and ROS‐free current, the results of this work can be further utilized. A challenge in the design of DCS experiments is the estimation of the electrode capacitance required to enable specific processes, such as the controlled release of ROS or the avoidance of irreversible reactions that generate toxic concentrations of stimulation by‐products. Throughout this work, we have shown which electrochemical reactions occur, and their rates are material dependent (e.g., O_2_ evolution only for Pt and SIROF). The voltage recorded during DCS informs about the onset of specific reactions if their activation potentials are known a priori. We here provide values for the activation potentials of O_2_ evolution and consumption and H_2_O_2_ evolution reactions for Pt, SIROF, SIROF ePEDOT, LIG, and LIG hPEDOT during DCS as they can be extracted by combining the information in Figure 4c,d,f,g. This data set can be easily extended when real‐time sensors suitable for measuring at the electrode's surface and sensitive to other reaction products (e.g., pH, metal oxides, hydrogen) become available. Knowing the activation potential, a voltage threshold can be set during a DC experiment to either end stimulation to avoid the generation or mark the start time of the controlled release of irreversible reaction by‐products. Our data show that the time to reach this threshold can be extended by increasing the capacitance of the stimulation electrodes with PEDOT coating. Both PEDOT formulations investigated here are easy to apply and suitable for micro‐ (ePEDOT) and macroelectrodes (ePEDOT and hPEDOT), enabling a longer time of nontoxic DCS. The time of capacitive‐like current for multiple DCS pulses decreases in consecutive pulses, because the first pulse leaves the electrode in a polarized state (Figure S11, Supporting Information). When the pre‐polarized electrode is further stimulated monophasically, the required activation potential for certain electrochemical reactions, for instance for H_2_O_2_ generation, is reached earlier. To obtain similar charge transfer dynamics in consecutive DCS a method to recover the electrode to its nonpolarized state (e.g., biphasic stimulation) is required. However, a full recovery of the electrode is only possible if the electrochemical reactions during the DCS are reversible.

The cytotoxicity of stimulation by‐products depends on their chemical composition, the cell type, and the temporal concentration gradient as for instance shown for H_2_O_2_.^[^ ^32^, ^34^ ^]^ With the help of lab on a chip (LoC), the correlation between time and amplitude of DCS, concentration of chemical by‐products, and apoptosis for various cell types could be studied. LoC offer the possibility to achieve results on application level, as gradients of the chemical and enzymatic buffers, and tissue perfusion can be modeled.^[^ ^72^, ^73^, ^74^ ^]^ A step further would be to integrate sensors in the stimulation device, which constantly monitor reaction by‐products in vivo. The concentration of stimulation by‐products in the tissue can further be influenced by coating the electrode with a material, which acts as barrier and hinders toxic ions to diffuse into the tissue.^[^ ^75^ ^]^ The performance of such composite‐based electrodes in the DCS regime is still unknown. Furthermore, we encourage other research groups to investigate if the recorded voltage during two‐electrode setup DCS can be simulated before the experiment takes place. Then, the time to reach a certain threshold voltage can be estimated without relying on such extensive experimentation as is presented here. This would greatly facilitate the planning of DC experiments in vitro and in vivo with better control of the concentration of stimulation by‐products. We think that the estimated capacitances, the measured concentrations, and the recorded voltages in this work are a starting point for such an investigation. Furthermore, we emphasize that modulation of biological processes, beyond the nervous system, using DCS mimicking the endogenous EFs is an application field yet in its infancy, and we encourage more groups to explore this new territory of bioelectronic medicine.

## 4 Conclusion

In this work, we show that the time of capacitive‐like current during DCS is enhanced by coating electrodes with PEDOT:PSS. We investigated the time dynamics of ORR and water electrolysis, which are irreversible electrochemical reactions. After coating LIG, which we show to be an effective electrocatalyst for the two‐electron ORR (i.e., resulting in the generation of H_2_O_2_), with hPEDOT, the time of capacitive‐like current is increased three times, and the H_2_O_2_ evolution is delayed. Coating SIROF with ePEDOT slightly enhances the electrode capacitance, resulting in a longer duration of capacitive‐like current. In addition, the ePEDOT coating slows the release of H_2_O_2_ and offers other reaction pathways that hinder O_2_ evolution, which was otherwise observed for pristine SIROF. Our results support using the recorded voltage to qualitatively determine the nature of the major contributing electrochemical reactions. Thus, a simplified evaluation method analyzing the slope of the recorded voltage can be used in future experiments to estimate the onset time of irreversible reactions. Future work should focus on developing suitable sensors to analyze other reaction products such as metal oxides, investigating the toxic concentrations of H_2_O_2_ and O_2_ in tissue, and simulating the voltage excursion during two‐electrode DCS to estimate the time of capacitive‐like current before experiments.

## 5 Experimental Section

### Electrode Materials

LIG electrodes were investigated in this work because they could be fabricated under a rapid prototyping regime.^[^ ^52^ ^]^ They were coated by spot casting to form a hydrogel PEDOT:PSS (hPEDOT) layer, which was shown to be superior for DCS in terms of charge storage capacity compared to electropolymerized ePEDOT on LIG.^[^ ^13^ ^]^ Fabrication of the electrodes is described in the previous work.^[^ ^52^ ^]^ In short, a laser engraver (Versa Laser 2.30, ULS Inc., AZ USA, 10.6 µm) was used to fabricate LIG electrodes. Kapton (Kapton 300HN, Dupont, USA) got carbonized during the rastering process (4.8 W, 15.2 mm s^−1^, 1000 PPI), forming the electrode sites and interconnections. Electrodes were coated with hPEDOT by spot casting. First, LIG was pretreated with air plasma (100 W, 5 min) (Femto, Diener Electronic, Germany) and soaked for 4 h in 10% (w/v) hexamethylenediamine (HMDA, H11696, Sigma–Aldrich). After washing with DI water and drying, the electrodes were coated with polyurethane (HydroMed D3, AdvanSource Biomaterials Corp., USA) in 90% (v/v) ethanol (1% w/v) using a dip‐coater (ND‐R Rotary Dip Coater, Nadetech Innovations, Spain). After 60 min on a hot plate at 80 °C, PEDOT dispersion was manually cast onto electrode sites using a micropipette. The PEDOT dispersion was a mixture of PEDOT solution (poly(3,4‐ethylenedioxythiophene)‐poly(styrenesulfonate), 1.3% (wt.) dispersion in H_2_O, Sigma–Aldrich, Germany) with dimethyl sulfoxide added to form a final solution of 15% (v/v). Then, electrodes were annealed on a hot plate overnight at 60 °C followed by 130 °C for 90 min. The interconnections were insulated using an acrylate‐based varnish (2 in 1 base and top coat super strong, Germany). For experiments shown in Figures 2 and 3, the electrodes were connected via alligator clips. The described process was unsuitable for forming microelectrodes because of manual spot casting and the resolution of the laser engraver. Therefore, for comparison and to serve a wide variety of applications other electrode materials already established in the laboratory especially for neurostimulation and ‐recording are included in this work.

Platinum (Pt) and sputtered iridium oxide (SIROF) electrodes were common materials in bioelectronics, especially to form microelectrodes.^[^ ^44^ ^]^ In previous work, the laboratory had shown that SIROF acts as an adhesion promoter for PEDOT and sustained DCS.^[^ ^12^, ^60^, ^76^ ^]^ For microelectrodes, manual spot casting of hPEDOT was challenging. Therefore, SIROF electrodes were coated with electropolymerized PEDOT (ePEDOT). SIROF and Pt electrodes were structured on polyimide (PI) substrate (U‐Varnish S polyimide, UBE Industries Ltd., Japan) in a cleanroom according to previously published protocols.^[^ ^60^, ^76^, ^77^ ^]^ In brief, Pt was sputtered (300 W RF, Univex 500, Leybold, Germany) to form electrode sites and interconnections. In the case of SIROF electrodes, a second layer of Iridium (Ir) was sputtered in a reactive oxygen atmosphere (100 W DC) only on the electrode sites. A second 5 um thick PI layer was spin‐coated onto the PI‐Pt or PI‐Pt‐SIROF stack. The electrode sites were opened using reactive ion etching in O_2_ atmosphere (STS Multiplex ICP, SPTS Technologies, United Kingdom). A wire was soldered to the solder spot of each electrode and subsequently insulated with the acrylate‐based varnish. The 20 mm^2^ designs from the previous work were used in this study.^[^ ^13^ ^]^ ePEDOT was coated potentiostatically (0.9 V, PGSTAT 204, Metrohm Autolab B.V., Germany) in a three‐electrode setup with stainless steel (≈20 cm^2^) as counter and Ag/AgCl electrode (Ag/AgCl, BASI, USA) as reference. The SIROF electrode was put in an aqueous solution containing 5 mg mL^−1^ sodium polystyrene sulfonate (Poly(sodium 4‐styrenesulfonate), Sigma–Aldrich) and 0.01 m 3,4‐ethylenedioxythiophene monomers (3,4‐ethylenedioxythiophene 97%, Sigma–Aldrich). The charge was monitored and cutoff once it reached 300 mC cm^−2^. The impedance spectrum of the electrodes in 1× PBS is shown in Figure S12 (Supporting Information).

### Electrochemical Surface Area

The anodic peak current ($I_{p}$) of CV at various scan rates (*v*) (1, 2, 4, 6, 8, 10, 25, 50, 75, 100, 200, 300 mV s^−1^) was assessed to inform about the electron‐transfer capabilities of the electrodes and to calculate the electrochemical surface area (ECSA). CV of Pt, LIG, and LIG hPEDOT electrodes were conducted in 10 mm K_3_Fe(CN)_6_ (potassium hexacyanoferrate(III) ACS reagent ≥ 99.0%, Sigma–Aldrich) solution. CV of SIROF and SIROF ePEDOT in 100 mm K_3_Fe(CN)_6_ solution. For the calculation of the ECSA clear positive peaks in the CVs were necessary. The concentration of K_3_Fe(CN)_6_ had to be increased for SIROF‐based electrodes, as the resulting current peak at the lower concentration was not satisfactory. It seems SIROF was less effective in the ferri/ferrocyanide reaction. All electrodes had an area of 0.2 cm^2^. The potential range was individually set for each electrode type to allow for monitoring of the reduction and oxidation peak in a three‐electrode setup, with stainless steel (≈20 cm^2^) as counter and Ag/AgCl electrode as reference. CV was recorded using an autolab potentiostat. The relation between $I_{p}$ and *v* can be described by the Randles–Sevcik equation^[^ ^42^ ^]^

(3) $$I_{p}=\left(2.69\cdot 10^{5}\right)\;n^{3/2}D^{1/2}\mathrm{Conc}\cdot \mathrm{ECSA}\cdot v^{1/2}$$

with diffusion coefficient for ferricyanide/ferrocyanide couple *D* = 6.70 × 10^−6^cm^2^ s^−1^, the molar concentration of ferricyanide *Conc* (10 or 100 mm) and the number of moles of electrons transferred per mole of the electroactive species (ferricyanide/ferrocyanide couple) *n* = 1.^[^ ^78^ ^]^ To calculate the ECSA, linear regression of *I_p_* versus *v* ^1/2^ was performed to obtain the slope *k* and calculate the ECSA with the following equation

(4) $$\mathrm{ECSA}=k/\left[\left(2.69\cdot 10^{5}\right)n^{3/2}D^{1/2}\mathrm{Conc}\right]$$

In the case of LIG hPEDOT the scan rate range to calculate ECSA was reduced to *v* < 25 mV s^−1^, to detect clear peaks in the CVs (see Figure S5 and Table S1 for least square fitting results, Supporting Information).

### Estimate Capacitance

CV at various scan rates (*v*) (1, 2, 4, 6, 8, 10, 25, 50, 75, 100, 200, 300 mV s^−1^) was performed in the same electrochemical cell in 1× phosphate‐buffered saline (PBS) (P3813, Sigma–Aldrich). The potential range for all electrodes was set to −0.6 to 0.9 mV. All electrodes had an area of 0.2 cm^2^ (see Figure 4h for an overview over methods).

Capacitance (*C^CV^*) from CV was calculated by

(5) $$C^{CV}=\int I\left(U\right)dU/\left(2v\Delta U\right)$$

with recorded current *I*(*U*), voltage *U*, and voltage window Δ*U*. For the Lindquist limited *C^b^*, the integration boundaries were changed, and Δ*U* was adapted accordingly. The threshold *b*‐value of 0.8 to calculate $C^{b}$ was motivated to include the pseudocapacitive Ir(III)/Ir(IV) surface reaction and because a spherical diffusion process yields a *b*‐value of 0.75.^[^ ^42^, ^67^ ^]^

Lindquist^[^ ^46^ ^]^ had described the relationship between current and scan rate according to Equation (1) with *a* and *b* as parameters. CVs from various scan rates were evaluated at each voltage to obtain *b* by performing linear regression with

(6) $$\log\left(I\right)=\log\left(a\right)+b\log\left(v\right)$$

The voltage window to calculate *C^b^* was adapted to only include the voltage range in which the anodic and cathodic current had a *b*‐value ≥ 0.8. Only fits at each voltage with an R‐value greater than 0.95 were considered (R‐values in Supplementary). For LIG hPEDOT the *I* versus *v* plot was only accurately fitted for *v* ≤ 50 mV s^−1^ due to the tilt of CVs at faster *v* (see tilted CVs in Figure S6, Supporting Information).

Trasatti^[^ ^48^ ^]^ formulated that the total voltametric charge (*q_t_*) consisted of the charge of the outer surface (*q_o_*) and the inner bulk of an electrode (*q_i_*)

(7) $$q_{t}=q_{o}+q_{i}$$

It was assumed that as sweep rate increases, *q_i_* which is diffusion‐controlled decreases with *v* ^−1/2^. On the other hand, the voltammetric charge should increase with *v* ^1/2^ for a decreased scan rate. These assumptions led to the following two equations

(8) $$q\;\left(v\right)=q_{o}+av^{-1/2}$$

and

(9) $$1/q\;\left(v\right)=1/q_{t}+bv^{1/2}$$

with *a* and *b* as fitting parameters. For *v* → ∞ and *v* → 0 it therefore applies

(10) $$q\;\left(v\to \infty \right)=q_{o}$$

(11) $$1/q\;\left(v\to 0\right)=1/q_{t}$$

In this work the electrode capacitance was used instead of charge, which is directly proportional

(12) C=q/ΔU

with Δ*U* as voltage window used. The capacitances calculated from the area enveloped by CVs (*v* ≤ 10 mV s^−1^) were used for linear regression toward the Equations (8) and (9) (see Figure S10 and Table S2,S3 for fitting parameters, Supporting Information).

Dunn^[^ ^47^ ^]^ described the current according to Equation (2) with parameters *k*_1_ and *k*_2_. *k*_1_ *v* is the capacitive current while *k*_2_ *v* ^1/2^ was the diffusion‐limited current. Linear regression was performed with

(13) $$\frac{I}{v^{\frac{1}{2}}}=k_{1}\;v^{1/2}+k_{2}$$

at each voltage in the CVs obtained at various scan rates to determine *k*_1_ and *k*_2_. Only fits at voltages with an R value above 0.95 were considered (see Figures S7,S8, Supporting Information).

### Amperometric Measurement of H_2_O_2_ and O_2_ Concentrations

A single one‐channel Free Radical Analyzer (World Precision Instruments, WPI) was used to quantify the concentrations of H_2_O_2_ and O_2_ during simultaneous chronopotentiometric recordings using a EmStat4S LR (low range) potentiostat (PalmSens). H_2_O_2_ and O_2_ concentrations were measured using a 2 mm wide H_2_O_2_ (ISO‐HPO‐2, polarization voltage: VH2O2 = + 450 mV, range: 10 nA, WPI) and a 2 mm O_2_ (ISO‐OXY‐2, polarization voltage: VO2 = + 700 mV, range: 1 µA, WPI) sensor, respectively. Sensor current signals were recorded using LabScribe Software (version 4.31) and a Lab‐Trax4/16 (WPI) data acquisition device. All measurements were conducted in 0.01 m phosphate‐buffered saline (PBS dissolved in DI water, pH 7.4, room temperature, P5368‐10PAK, Sigma–Aldrich). The setup depicted in Figure 4a consisted of a transparent plastic box that was filled with 100 mL 1× PBS. All electrodes were glued to a flat, thin plastic substrate with double‐sided adhesive tape. The substrates with the electrodes were then fully immersed in PBS and attached to the bottom of the box with a polyimide adhesive tape. Before immersion into PBS the contact points of all LIG and hPEDOT LIG electrodes were connected to a wire using a silver conductive epoxy adhesive (MG chemicals, 8331S, DigiKey part number 473‐1178‐ND) followed by a final seal using a non‐conductive epoxy adhesive. Calibration of the H_2_O_2_ and O_2_ sensors was also performed in PBS solution, which had to be stirred continuously. The 5‐point calibration of the H_2_O_2_ sensor was carried out by placing the sensor tip into 40 mL of fresh PBS solution and sequentially adding 165, 330, 750, and 750 µL of a 1 mm H_2_O_2_ stock solution in DI water. For the O_2_ sensor, a 2‐point calibration was conducted using a calibration bottle (WPI). The current signal of the O_2_ sensor was first recorded in air‐saturated PBS solution (21% O_2_) followed by the current signal while purging the bottle with N_2_ (0% O_2_).

Measured concentrations and recorded voltages were smoothed by a moving average function with a 5 s window (see Figure S13–S17 for raw data plots, Supporting Information). *t*_1_(*Conc*) and *t*_50_(*Conc*) were the times when the measured concentration had been above the threshold over a summed period of 5 s with thresholds being 1% and 50% of the maximum/minimum reached concentration, respectively. The capacitive‐current time was defined as the time when the first derivative of the recorded voltage during H_2_O_2_ monitoring falls below 0.0002. The threshold was chosen to lay in the vertex of the upward recorded voltage curve. All data were analyzed using custom Python scripts.

## Conflict of Interest

The authors declare no conflict of interest.

## Author Contributions

L.M. and O.A. contributed equally to this work. S.S. conceived the original idea and planned experiments. L.M. carried out the CV experiments. O.A. carried out ROS measurements. L.M. fabricated SIROF and Pt‐based electrodes. S.S. fabricated the LIG‐based electrodes. L.M. processed the experimental data and performed analysis. L.M., J.L., and M.A. designed the figures. L.M. and O.A. wrote the manuscript. M.A. supervised the project supporting data analysis and writing. All authors provided critical feedback and helped shape the research, analysis, and manuscript.

## Supporting information

Supporting Information

## Data Availability

The data that support the findings of this study are available from the corresponding author upon reasonable request.
