# Supplementary material for: Bioelectronic Direct Current Stimulation at the Transition Between Reversible and Irreversible Charge Transfer
Source: Adv Sci (Weinh). 2024 Mar 9;11(27):2306244. doi: 10.1002/advs.202306244 (PMC11251568; doi:10.1002/advs.202306244)
Supplement: Supplementary file 1 — Supporting Information [file ADVS-11-2306244-s001.pdf]

ADVANCED SCIENCE
Open Access

Supporting Information

for *Adv. Sci.*, DOI 10.1002/advs.202306244

Bioelectronic Direct Current Stimulation at the Transition Between Reversible and Irreversible Charge Transfer

Lukas Matter, Oliya S. Abdullaeva, Sebastian Shaner, José Leal and Maria Asplund**

Supporting Information

Bioelectronic Direct Current Stimulation at the Transition Between Reversible and Irreversible Charge Transfer

Lukas Matter, Oliya S. Abdullaeva, Sebastian Shaner, José Leal, Maria Asplund

Like O_2 and H_2O_2, the pH is a marker for ORR and water electrolysis (see reactions in Table 1). However, miniature pH sensors with integrated reference electrode and faraday shielding were not commercially available to the time of this work which is why pH is not integrated into the main paper. Polyaniline (PANI) is a polymer that changes color depending on the pH of the surrounding electrolyte (dark blue = basic, light green = acidic). We wanted to explore the capacity of PANI to detect pH changes during DCS in two-electrode setup with the 20 mm^2 electrode used throughout this work. PANI was made from chemical oxidative polymerization on top of 1 mm thick acrylic sheets. We refer the reader to Ref. [1] Sec. 5.4 for a detailed description of the coating process. As shown in Figure S1 the PANI coated acrylic sheet and the electrodes were put in a customized holder device, filled with 5 ml electrolyte solution. In earlier experiments we identified that the applied current density of 10 μAcm^{-2} over 1 h did not surpass the buffering capacity of 5 ml 1xPBS, which presents the reason why we used a saline solution with similar ionic content as 1xPBS but without the phosphate buffer. Over the time of DCS slight changes in the color of PANI can be observed, turning more dark blue at the cathode and more light green at the anode (Figure S1). This is expected, since ORR and water reduction consume H^+ and generate OH^- lowering the pH at the cathode, but water oxidation releases H^+ at the anode. However, we observed that calibration of this method is difficult since the coated PANI without applying stimulation already had different colors for different experiments. In Ref. [1] we show that coating a micro channel instead of a big piece of acrylic and applying DCS through the microchannel mitigates this challenge. This was not relevant for this work, because we were interested in pH changes close to the electrode`s surface. We also noted that the applied current density only slightly shifts the pH of the unbuffered solution, leading to small color differences which are also spatially distributed. The spatially distribution make the analysis of the pictures difficult since a color average over half of the acrylic sheet would need to be calculated and assigned to the corresponding electrode. We increased the applied current density for SIROF ePEDOT by a factor of 100 and Figure S1 clearly shows that the pH changes significantly during the stimulation. For the increased applied current density PANI has a good sensitivity and could be useful to determine spatial pH changes.

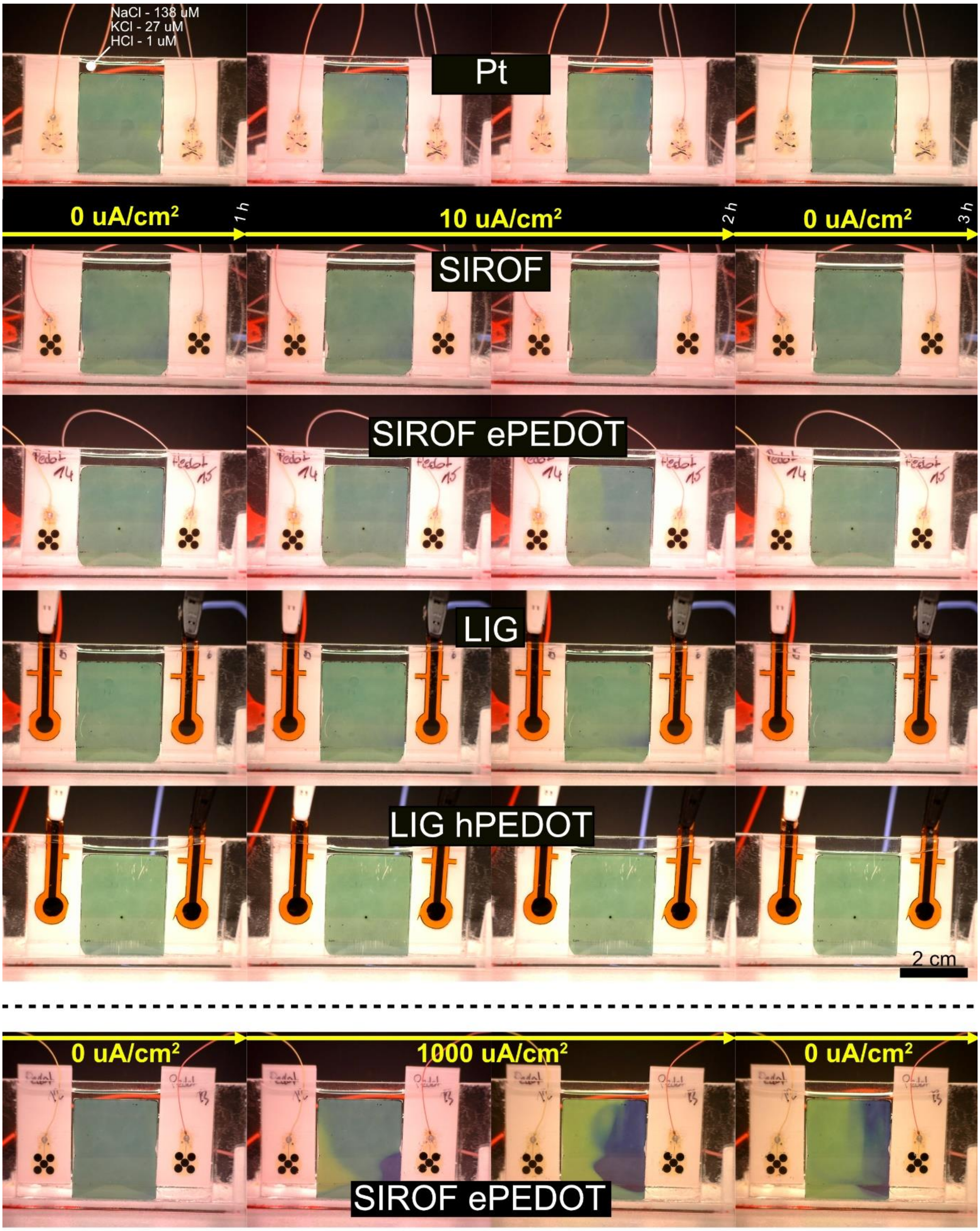

Figure S1. PANI coated acrylic sheets were used to identify relative pH changes during DCS. The stimulation protocol consisted of 1 h no stimulation, 1 h stimulation followed by 1 h no stimulation (anode left, cathode right). Pictures were taken every minute, the four pictures in the figure refer to 1, 1.5, 2 and 3 h. The color changes in PANI are only small for 10 μAcm^{-2}. By increasing the current density by a factor of 100, color changes are easily observed meaning pH changes significantly.

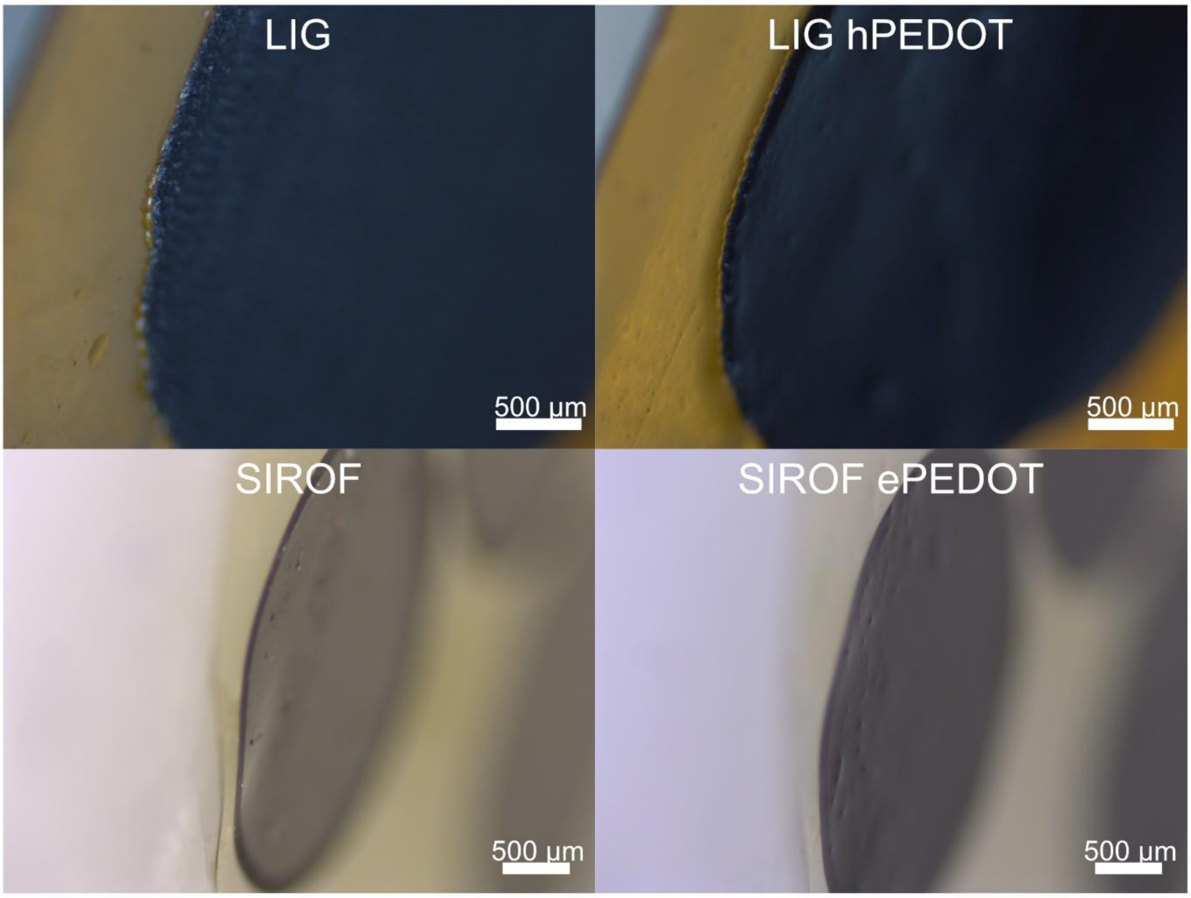

Figure S2. Pictures of tilted SIROF and LIG electrodes. PEDOT coating is visible for both base electrodes as the dark coating above the electrode surface.

Electrochemical surface area

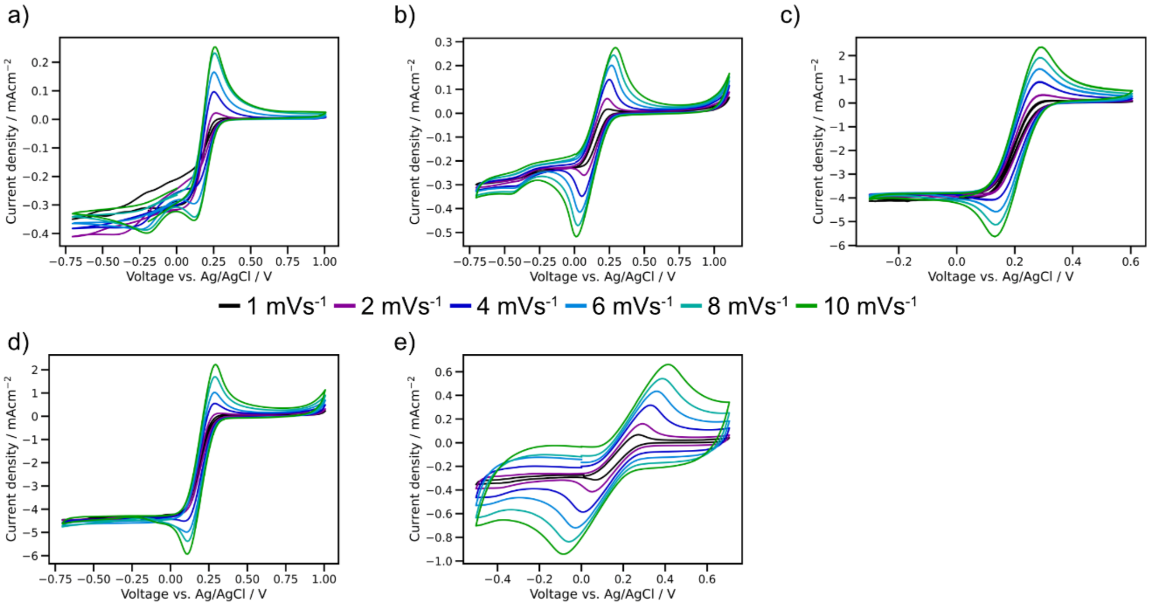

Figure S3. a) CVs of Pt in 10 mM $K_3Fe(CN)_6$. b) CVs of LIG in 10 mM $K_3Fe(CN)_6$. c) CV of SIROF in 100 mM $K_3Fe(CN)_6$. d) CV of SIROF ePEDOT in 100 mM $K_3Fe(CN)_6$. e) CV of LIG hPEDOT in 10 mM $K_3Fe(CN)_6$.

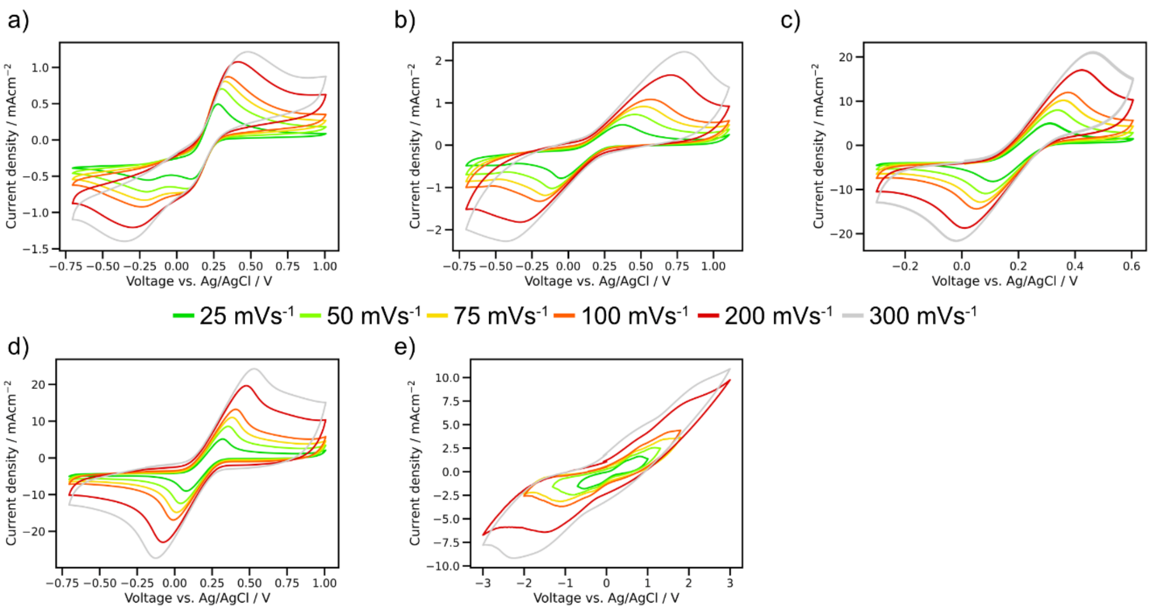

Figure S4. a) CVs of Pt in 10 mM $K_3Fe(CN)_6$. b) CVs of LIG in 10 mM $K_3Fe(CN)_6$. c) CV of SIROF in 100 mM $K_3Fe(CN)_6$. d) CV of SIROF ePEDOT in 100 mM $K_3Fe(CN)_6$. e) CV of LIG hPEDOT in 10 mM $K_3Fe(CN)_6$.

The anodic peak is not clearly expressed in Figure S4e which presents the reason why for the calculation of the effective surface area of LIG hPEDOT the scan rate range was reduced to CVs seen in Figure S3e.

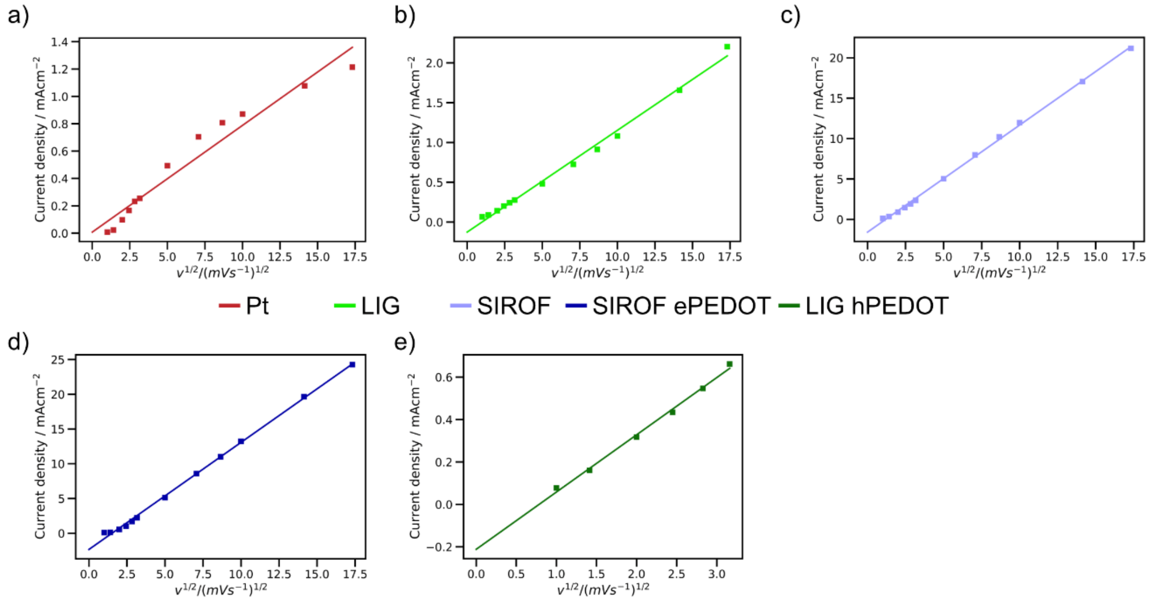

Figure S5. Linear regression to calculate effective surface area from Randles-Sevcik equation of a) Pt, b) LIG, c) SIROF, d) SIROF ePEDOT and e) LIG hPEDOT.

Table S1. Linear regression parameters. Note that SIROF and SIROF ePEDOT were measured in 100 mM $K_3Fe(CN)_6$ whereas Pt, LIG and LIG hPEDOT in 10 mM.

**Material**	**Intercept**	**Slope**	**R-value**
**Pt**	1.44 e-6	1.56 e-5	0.98
**SIROF**	-3.16 e-4	2.65 e-4	0.99

**SIROF ePEDOT**	-4.68 e-4	3.08 e-4	0.99
**LIG**	-2.54 e-5	2.56 e-5	0.99
**LIG hPEDOT**	-4.23 e-5	5.39 e-5	0.99

Analysis of cyclic voltammograms

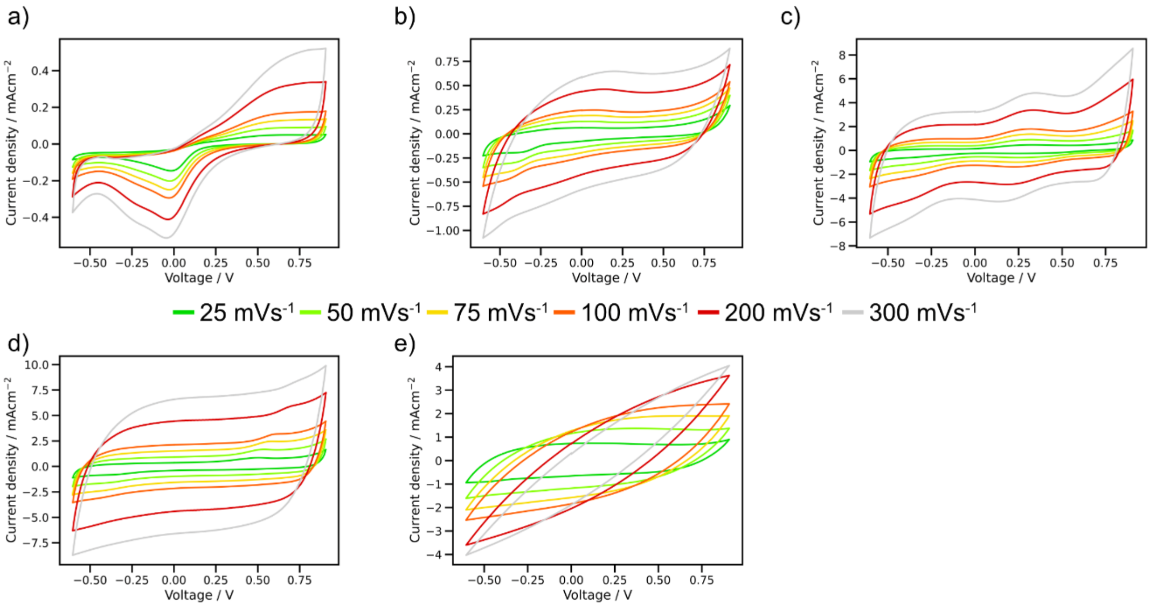

Figure S6. a) CVs of Pt. b) LIG. c) SIROF. d) SIROF ePEDOT. e) LIG hPEDOT. All in 1x PBS.

Estimation of electrode capacitance

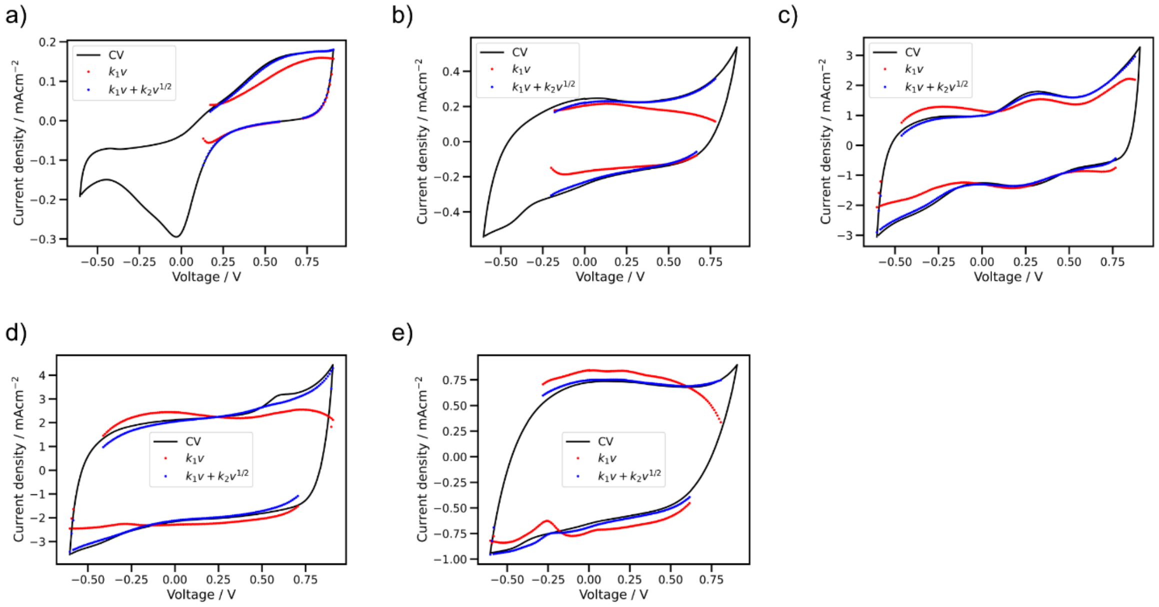

Figure S7. CVs with capacitive part of the current from Dunn method for a) Pt, b) LIG, c) SIROF, d) SIROF ePEDOT all at 100 mVs^{-1} and LIG hPEDOT at 25 mVs^{-1}. Only points with an R-value $\geq$ 0.95 were considered.

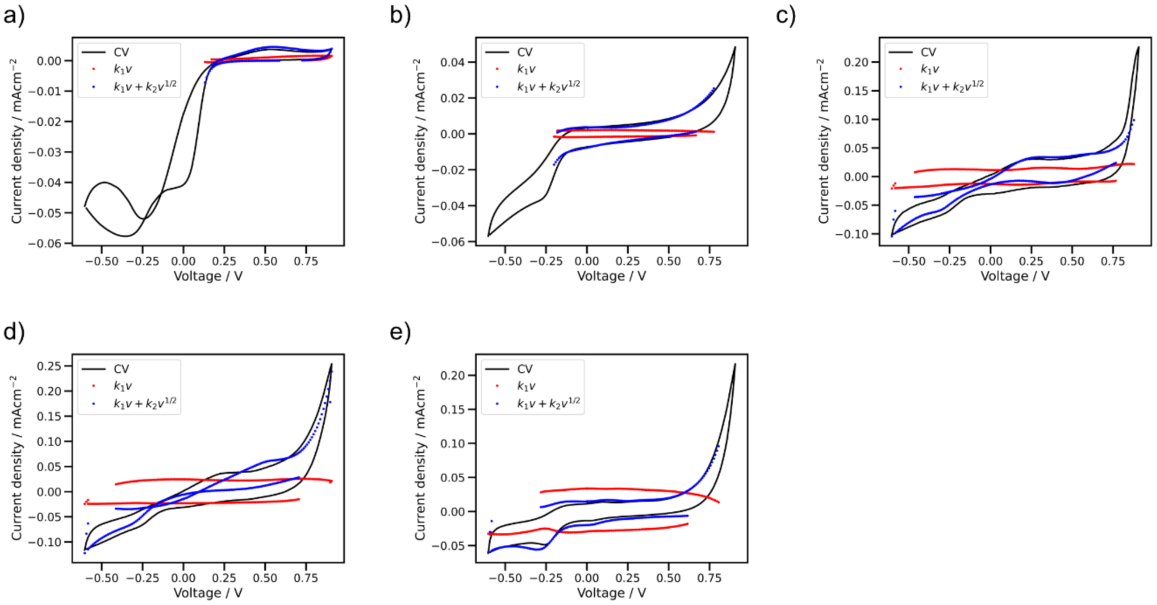

Figure S8. CVs with capacitive part of the current from Dunn method for a) Pt, b) LIG, c) SIROF, d) SIROF ePEDOT and e) LIG hPEDOT all at 1 mVs^{-1}. Only points with an R-value ≥ 0.95 were considered.

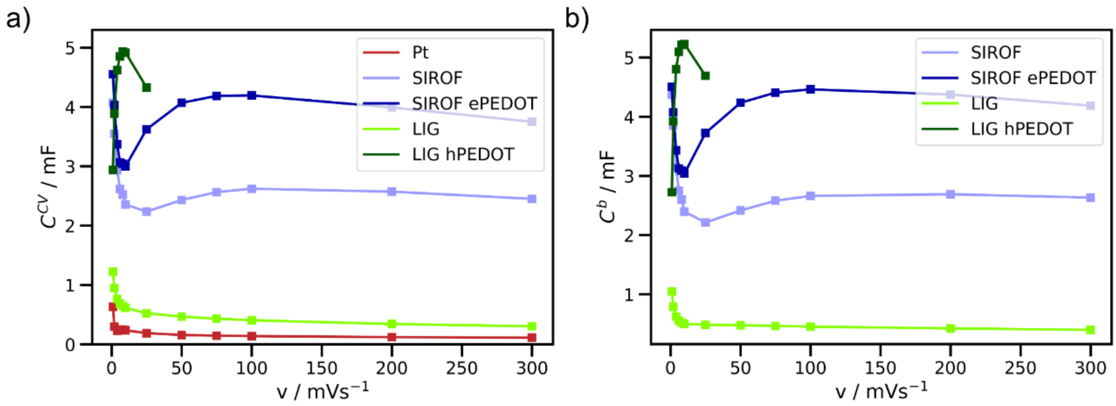

Figure S9. a) Capacitance from charge of CV. b) Capacitance from charge of CV for b ≥ 0.8 according to Lindquist method.

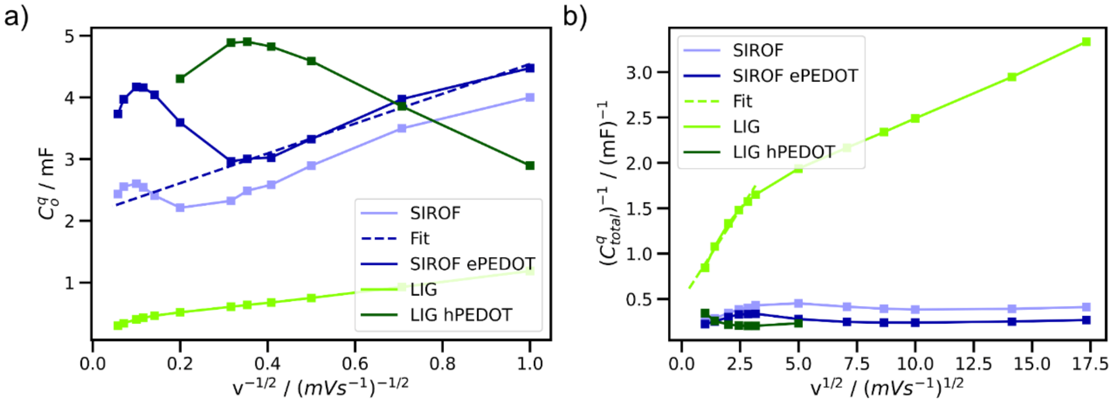

Figure S10. a) $C_o^q$is the y-intercept of C_o^q vs. $v^{-1/2}$ plot according to Trasatti method. b) $1/C_{total}^q$is the y-intercept of $1/C_{total}^q$ vs. $v^{1/2}$ plot according to Trasatti method.

Table S2. Linear regression parameters for Trasatti between 1 and 10 mVs^{-1} to calculate C_o^q.

**Material**	**Intercept**	**Slope**	**R-value**
**SIROF**	1.66 e-3	7.63 e-5	0.992
**SIROF ePEDOT**	2.13 e-3	7.63 e-5	0.990
**LIG**	3.27 e-4	2.71 e-5	0.999
**LIG hPEDOT**	6.11 e-3	-1.01 e-4	0.998

Table S3. Linear regression parameters for Trasatti between 1 and 10 mVs^{-1} to calculate $1/C_{total}^q$.

**Material**	**Intercept**	**Slope**	**R-value**
**SIROF**	1.66 e2	2.75 e3	0.994
**SIROF ePEDOT**	1.63 e2	2.03 e3	0.982
**LIG**	4.88 e2	1.26 e4	0.990
**LIG hPEDOT**	3.86 e2	-2.27 e3	0.901

Pulsed direct current stimulation

Certain biological applications require consecutive DCS. For instance, pulse widths of 15 min (oscillating field stimulation) were found to guide and support axonal regeneration after spinal cord injury [2]. DC is a term typically used to describe a steady, continuous current (*i.e.*, not a time-limited pulse). In the practical context of bioelectronic stimulation, impulses are nevertheless always confined to a certain duration or stimulation time. Thus, in this work the term DCS describes a time-limited current, albeit of a much longer pulse duration (minutes to hours) compared to what is typical in neurostimulation (a few hundred µs). To determine if PEDOT coating delays the onset of H_2O_2 generation during consecutive DCS we repeated the amperometric measurements of H_2O_2 while simultaneously applying four 15 min pulses (10 $\mu A/cm^2$) with 15 min inter-pulse period in the same two-electrode setup as shown in Figure 4a. The measured H_2O_2 concentrations and the corresponding voltage recordings are summarized in Figure S11. While the ePEDOT and hPEDOT coatings delay the onset of H_2O_2 generation during the first pulse, the generation of H_2O_2 occurs earlier during the three consecutive pulses. Furthermore, during the last three pulses, slightly higher concentrations of H_2O_2 are generated by all electrode materials except Pt compared to the first pulse. By analyzing the corresponding voltage recordings shown in Figures S11b and S11d, these observations can be explained. The electrodes are polarized during the first pulse and are not able to recover to a non-polarized state during the 15 min inter-pulse period, as evidenced by the increasing voltage values at the start of each consecutive pulse. Therefore, the electrodes reach the voltage required for H_2O_2 generation earlier during the consecutive pulses than during the first pulse.

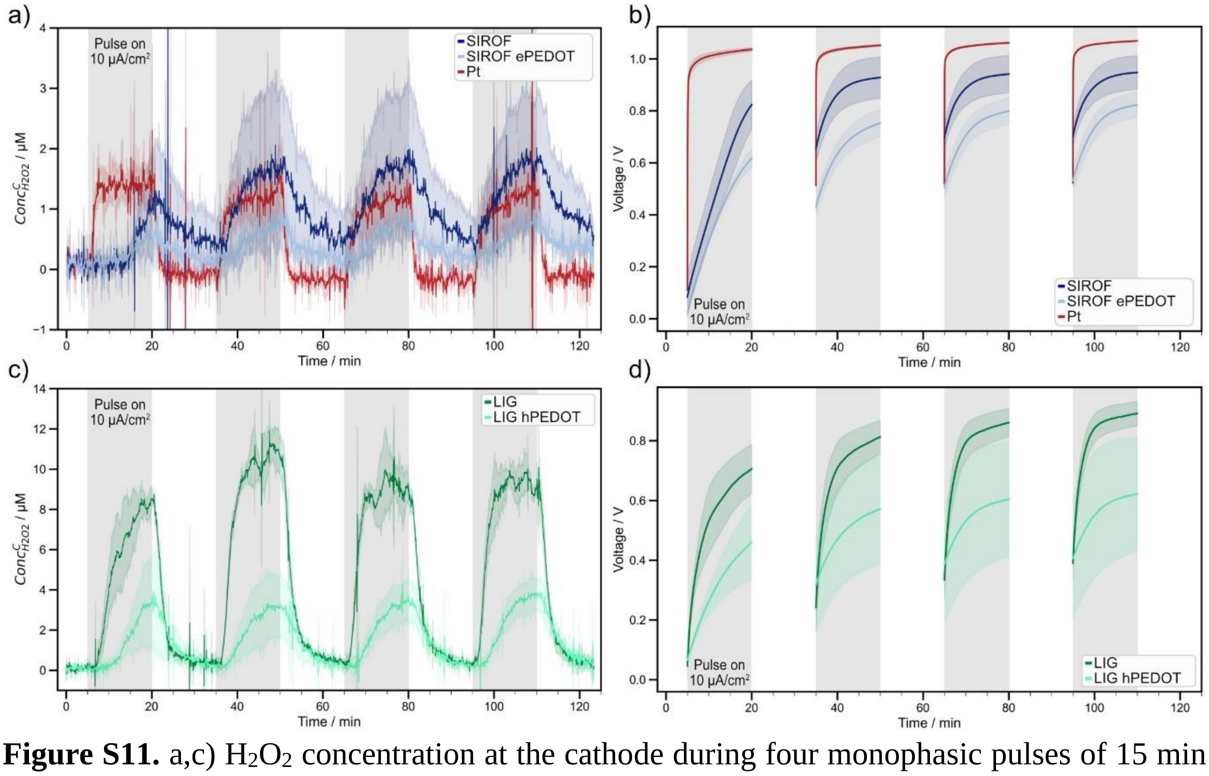

Figure S11. a,c) H_2O_2 concentration at the cathode during four monophasic pulses of 15 min with 15 min between pulses. c,d) Voltage excursion during the pulsing in 2-electrode setup.

Impedance spectroscopy

EIS was performed using an Autolab potentiostat (PGSTAT 204, Metrohm Autolab B.V., Filderstadt, Germany) in 3 electrode setup with stainless steel (~ 20 cm^2) as counter and Ag/AgCl electrode (Ag/AgCl, BASI, USA) as reference. As electrolyte 0.01 M phosphate-buffered saline (PBS, Sigma Aldrich, USA) was used. All electrodes had an area of 0.2 cm^2. Before EIS the electrode underwent 5 CV cycles (-600 to 900 mV, 100 mV/s). EIS was performed with a 100 mV sine amplitude with 5 points per decade between 0.1 Hz and 100 kHz.

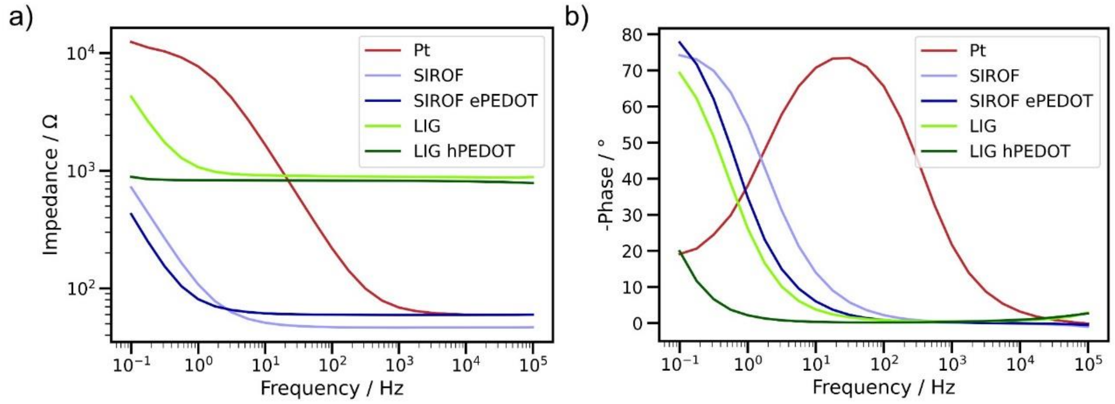

Figure S12. a) Impedance of the investigated electrode materials. b) Phase of the investigated electrode materials

Measurement of H_2O_2 and O_2 during direct current stimulation

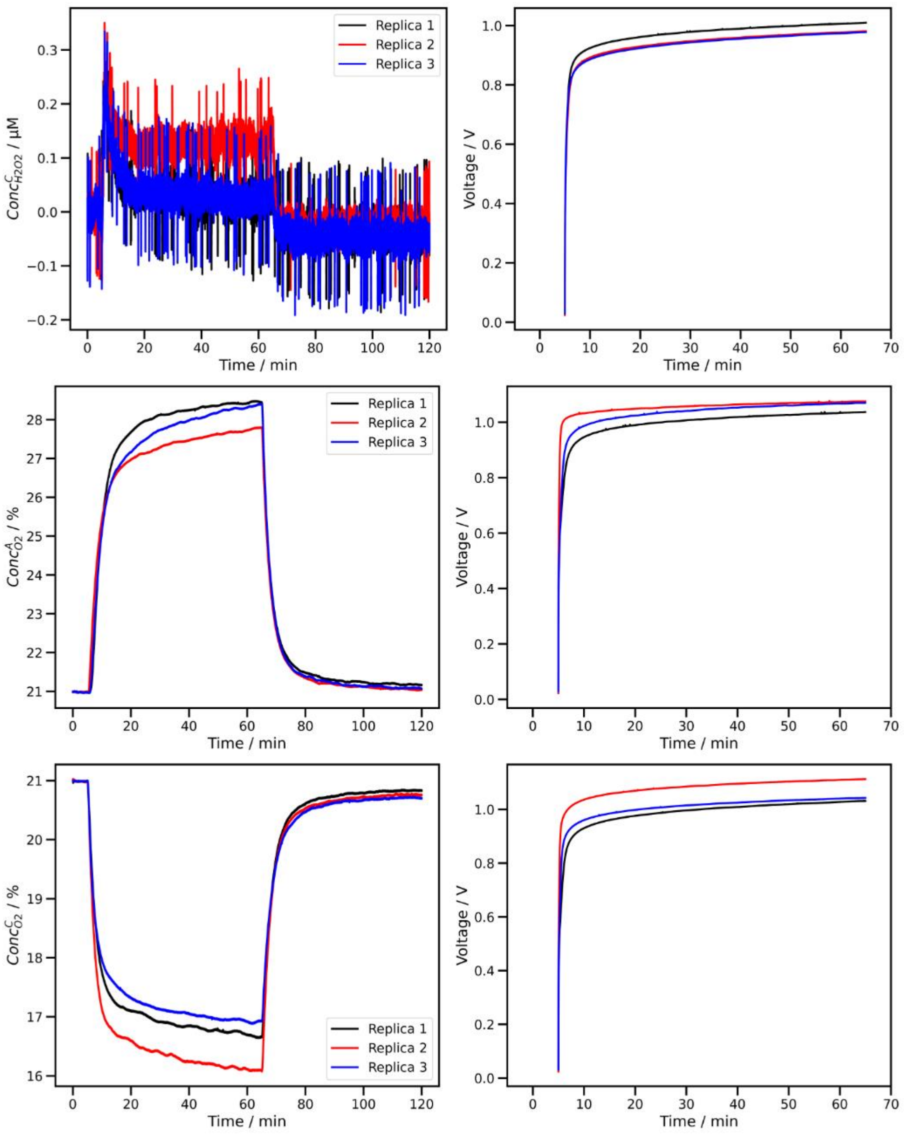

Figure S13. Measured concentration and recorded voltage for Pt.

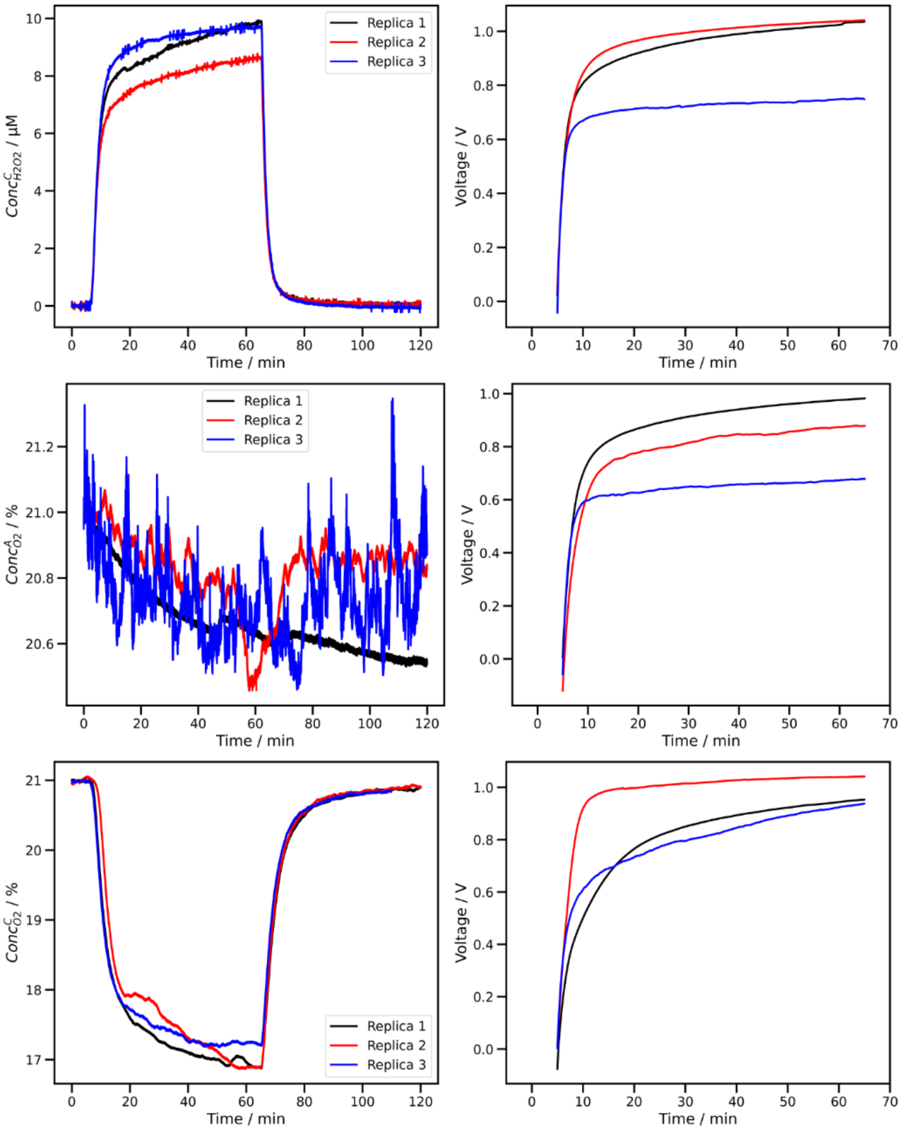

Figure S14. Measured concentration and recorded voltage for LIG.

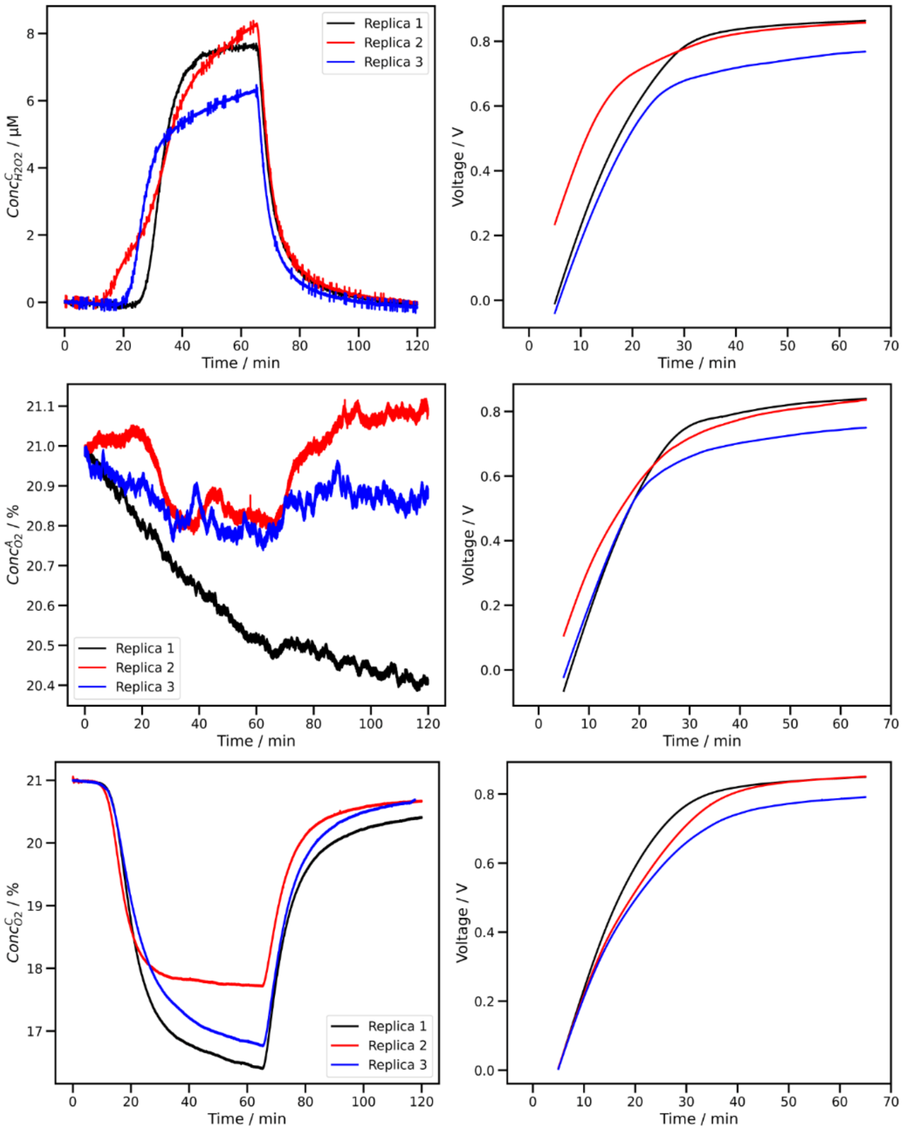

Figure S15. Measured concentration and recorded voltage for LIG hPEDOT.

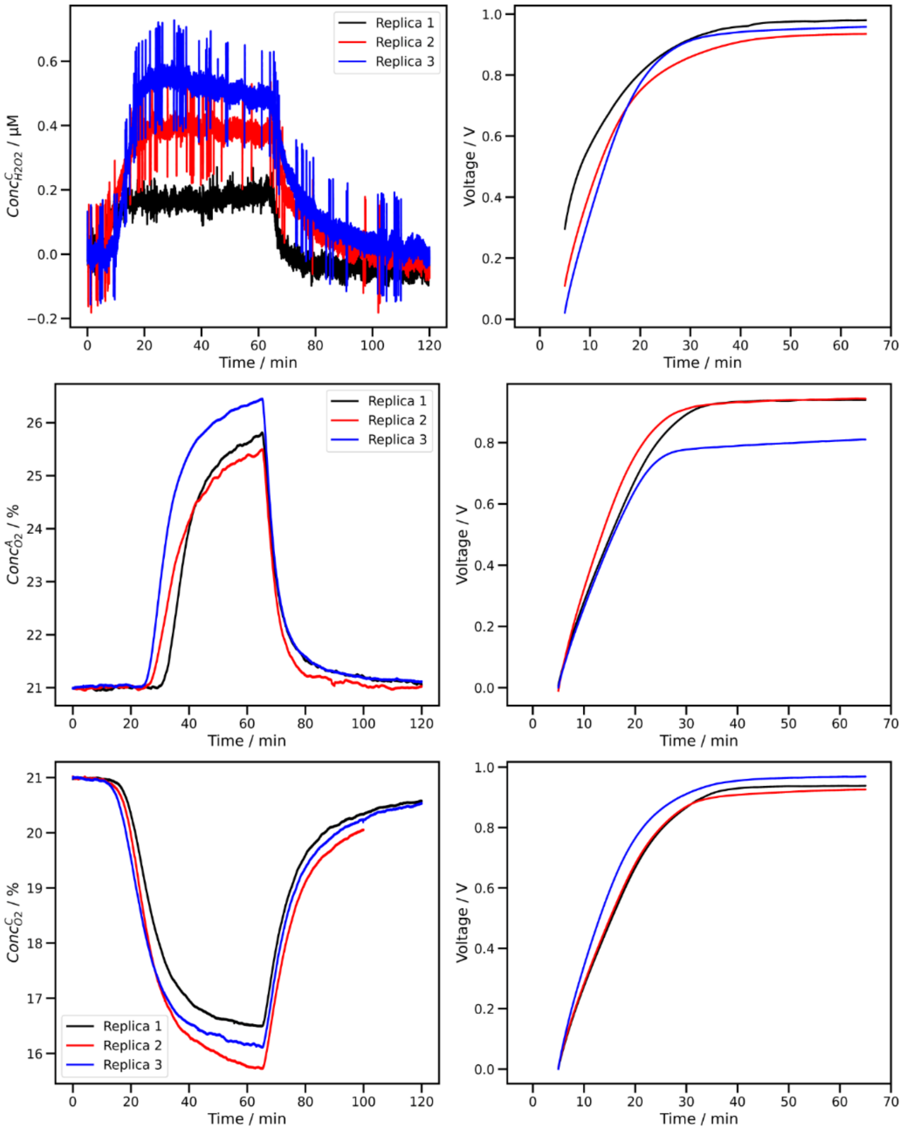

Figure S16. Measured concentration and recorded voltage for SIROF.

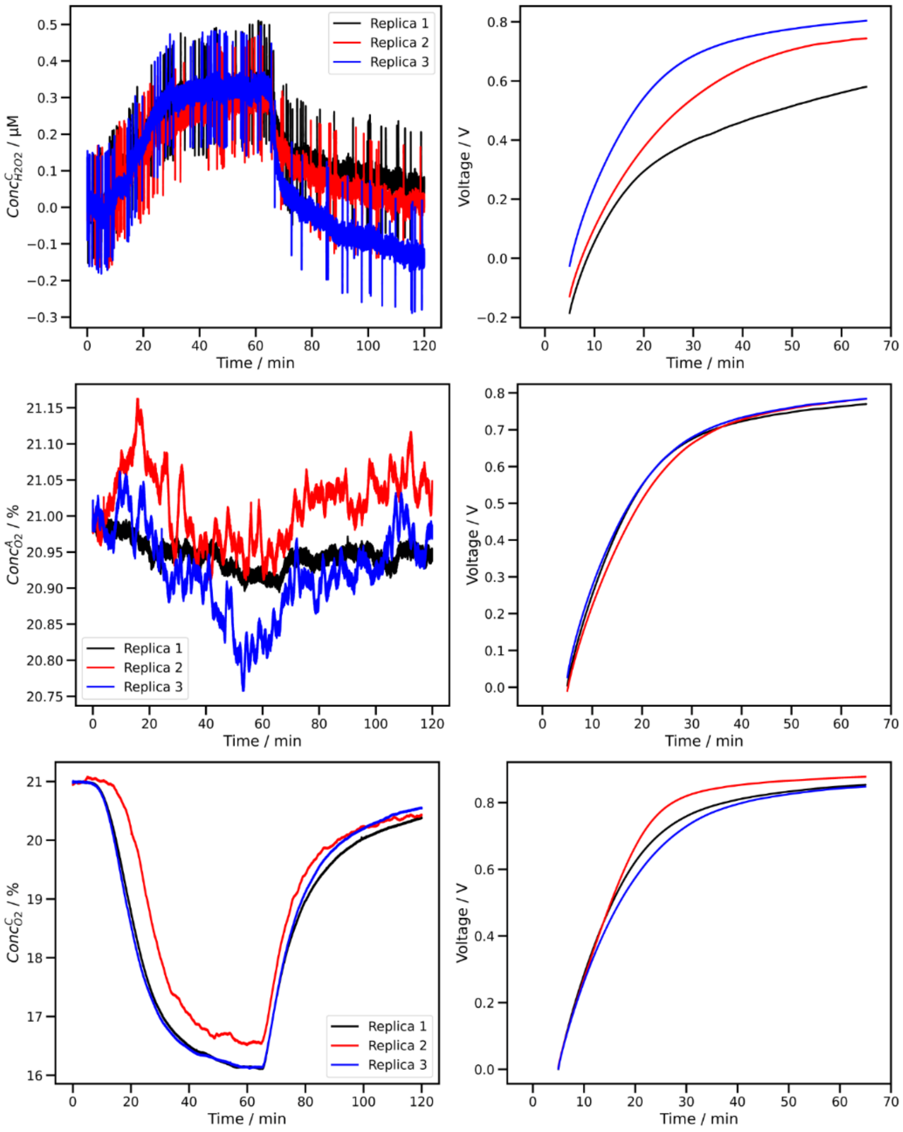

Figure S17. Measured concentration and recorded voltage for SIROF ePEDOT.

References

[1] S. Shaner, A. Savelyeva, A. Kvartuh, N. Jedrusik, L. Matter, J. Leal, M. Asplund, *Lab Chip* **2023**, *23*, 1531.

[2] A. S. Jack, C. Hurd, J. Martin, K. Fouad, *J Neurotrauma* **2020**, 37, 1933.
